# Analysis of transcripts differentially expressed between fruited and deflowered ‘Gala’ adult trees: a contribution to biennial bearing understanding in apple

**DOI:** 10.1186/s12870-016-0739-y

**Published:** 2016-02-29

**Authors:** B. Guitton, J. J. Kelner, J. M. Celton, X. Sabau, J. P. Renou, D. Chagné, E. Costes

**Affiliations:** INRA, UMR AGAP, CIRAD-INRA-SupAgro, AFEF team (Architecture et Fonctionnement des Espèces Fruitières) TA 108/03, Avenue Agropolis, 34398, Montpellier, CEDEX 5 France; ICRISAT, Samanko station, BP320, Bamako, Mali; CIRAD, UMR AGAP, CIRAD-INRA-SupAgro, TA 108/03, Avenue Agropolis, 34398, Montpellier, CEDEX 5 France; SupAgro, UMR AGAP, CIRAD-INRA-SupAgro, AFEF team (Architecture et Fonctionnement des Espèces Fruitières) TA 108/03, Avenue Agropolis, 34398, Montpellier, CEDEX 5 France; INRA, UMR1345 IRHS, Institut de Recherche en Horticulture et Semences, AgroCampus-Ouest-INRA- QUASAV, Bretagne-Loire University, 49071 Beaucouzé, France; The New Zealand Institute for Plant & Food Research Limited, Private Bag 11600, Palmerston North, 4442 New Zealand

**Keywords:** *Malus x domestica*, Floral induction, Floral inhibition, Microarray, qRT-PCR, Alternate bearing

## Abstract

**Background:**

The transition from vegetative to floral state in shoot apical meristems (SAM) is a key event in plant development and is of crucial importance for reproductive success. In perennial plants, this event is recurrent during tree life and subject to both within-tree and between-years heterogeneity. In the present study, our goal was to identify candidate processes involved in the repression or induction of flowering in apical buds of adult apple trees.

**Results:**

Genes differentially expressed (GDE) were examined between trees artificially set in either ‘ON’ or ‘OFF’ situation, and in which floral induction (FI) was shown to be inhibited or induced in most buds, respectively, using qRT-PCR and microarray analysis. From the period of FI through to flower differentiation, GDE belonged to four main biological processes (i) response to stimuli, including response to oxidative stress; (ii) cellular processes, (iii) cell wall biogenesis, and (iv) metabolic processes including carbohydrate biosynthesis and lipid metabolic process. Several key regulator genes, especially *TEMPRANILLO* (*TEM*), *FLORAL TRANSITION AT MERISTEM* (*FTM1*) and *SQUAMOSA PROMOTER BINDING PROTEIN-LIKE* (*SPL*) were found differentially expressed. Moreover, homologs of *SPL* and Leucine-Rich Repeat proteins were present under QTL zones previously detected for biennial bearing.

**Conclusions:**

This data set suggests that apical buds of ‘ON’ and ‘OFF’ trees were in different physiological states, resulting from different metabolic, hormonal and redox status which are likely to contribute to FI control in adult apple trees. Investigations on carbohydrate and hormonal fluxes from sources to SAM and on cell detoxification process are expected to further contribute to the identification of the underlying physiological mechanisms of FI in adult apple trees.

**Electronic supplementary material:**

The online version of this article (doi:10.1186/s12870-016-0739-y) contains supplementary material, which is available to authorized users.

## Background

In higher plants, the transition toward flowering occurs under the control of environmental and endogenous stimuli which are categorized in several partially overlapping genetic pathways [60, 103]. In *Arabidopsis thaliana*, the final outputs of all pathways has been shown to converge on a limited number of flower-promoting proteins in the meristem, especially those encoded by the genes *FLOWERING LOCUS T* (*FT*) and *SUPPRESSOR OF OVEREXPRESSION OF CONSTANS 1* (*SOC1*) [41, 58, 82]. These proteins activate floral meristem identity genes such as *LEAFY* (*LFY*) and *APETALA1* (*AP1*), which in turn activate floral homeotic genes responsible for floral organ differentiation [48, 58, 80]. However, floral transition can be delayed by floral repressors such as *TERMINAL FLOWER1* (*TFL1*) and *BROTHER of FT and TFL1* (*BFT*), which repress *LFY* and *AP1* [10, 60, 61, 96, 117]. Moreover, regulations involving *FT* repressors have been highlighted in *Arabidopsis* grown under long day (LD) or in non-inductive conditions. Floral repressors, especially *SHORT VEGETATIVE PHASE* (*SVP*), *FLOWERING LOCUS C* (*FLC*), and *TEMPRANILLO* (*TEM1* and *TEM2*), have been shown to interact with the *CONSTANS* (*CO*)-*FT* genetic pathway to determine the optimal timing of floral transition depending on day length and temperature [83, 91]. *TEM1* and *TEM2* can regulate *FT* expression to an extent that changes during development [45, 86]. Also, two highly conserved microRNAs (miRNAs), *miR156* and *miR172* [76, 112] target *SQUAMOSA PROMOTER BINDING PROTEIN-LIKE* (*SPL*) family transcription factors, which promote the transition from the juvenile to the adult phase, in *Populus* [110] and key floral repressors belonging to the *AP2-like* transcription factor family genes [118], respectively. Other age-dependent pathways involve carbohydrates directly [108] or in conjunction with photoperiod [62]. In addition, the redox status of the plant, which is linked to its perception of environment and is involved in the control of plant growth and development through the reprogramming of metabolism, might be involved in the control of floral transition [8, 49].

Although there are some fundamental differences in flowering biology between annual and perennial plants, knowledge acquired in model plants can be used for examining control of flowering in perennial species [100]. Because of their great economic importance for the horticultural industry, numerous studies have contributed to decipher the flowering process in apple and in the model tree species poplar (for review see [12, 35, 56, 111]). A set of genes showing sequence similarity with known flowering genes in *Arabidopsis* has been shown to have similar function in apple tree [26, 28, 36, 44, 50–55, 67, 68, 94, 95, 105, 107, 115, 116]. Moreover, the tree juvenile phase has been successfully reduced by knocking-out or over-expressing floral or floral-repressor genes (e.g. [99]) and the temporal expression of 10 flowering genes in apple apical buds suggests that they may play central roles in FI and flower organ development [36]. However, these studies focused on aspects of flowering related to the transition from juvenile to mature trees and to seasonal control of flowering date.

Although flowering is recurrent in perennial plants over consecutive years, it only occurs in a subset of buds in each growth cycle [111]. In apple, inflorescences may form in terminal buds of all axes. They remain preformed in dormant mixed buds that include leaf primordia followed by the inflorescence and the first primordia of an axillary shoot ([21, 31]). The short preformed growth unit carrying the inflorescence is usually called a bourse and its axillary sympodial shoot, the bourse shoot. Even though this may differ among varieties, histological observations and mRNA transcript analysis agree that FI occurs in apical buds of short shoots between 39 and 53 days after full bloom (DAFB), and that morphological changes corresponding to flower bud initiation start approximately at 60 DAFB [13, 23, 29, 35, 53]. However, the floral transition occurs only once the vegetative development of the axis is completed, the timing of which varies greatly depending on its type and position within the tree [19]. Whatever their within-tree situation, FI and flower bud initiation occur during spring or summer, i.e. in long days (LD) and under high temperatures, while fruit development is ongoing. Fruit and vegetative growth are generally considered to be negatively correlated [47] and FI to be strongly inhibited by concurrent fruiting. This may lead to biennial bearing, this term referring to trees having an irregular crop load from year to year. ‘ON’ years characterized by significant production are followed by ‘OFF’ years, characterized by low production. The low production in ‘OFF’ years usually results from a lack of flower formation rather than a poor fruit-set [47, 69, 89]. Therefore, biennial bearing, which is a problem observed in many fruiting trees, appears to be a problem of FI rather than floral differentiation.

Hence, floral transition between consecutive seasons in fruit tree species is still poorly understood and the physiological processes triggering or inhibiting flower formation remain to be identified. In mandarins, the genes and proteins profiles have been compared between fruited and defruited trees, this demonstrating that proteins involved in primary metabolism and redox state were differentially expressed in leaves depending on fruit load [70]. In addition, photosynthetic genes and calcium-dependent IAA transport were induced rapidly after defruiting treatment and in ‘OFF’ citrus trees [87]. In mango, the autonomous GA pathways appear to be involved in both biennial bearing and flowering control [71, 77].

In the present study, our objective was to investigate biological processes that are involved in FI in apical buds of adult apple trees. To achieve this, we manipulated trees for being in either on ‘ON’ or ‘OFF’ status and we assumed their apical buds to be more likely under inhibition or induction conditions, respectively. We then studied the effect of the presence of fruits on the differential expression of genes involved in floral transition in apical buds of bourse shoots in those adult apple trees, using a microarray analysis. Quantitative Reverse Transcription PCR (qRT-PCR) was also performed to study the relative expression of key flowering genes between ‘ON’ and ‘OFF’ trees during the critical periods for FI and floral differentiation and to validate the microarray. This study highlighted processes that are differentially regulated between buds more likely to flower than others within adult apple trees. How many of these processes actually regulate flowering remains an open question that will require further investigations.

## Methods

### Plant material

Sixteen year old trees of *Malus* x *domestica* ‘Gala’ (clone ‘Galaxy’) were grown at the CEHM experimental station (Centre Experimental Horticole de Marsillargues) (France), according to normal commercial practices. A set of five trees was set in the ‘OFF’ situation by completely hand deflowering the trees at full bloom. Another set of five trees with no manual or chemical thinning applied was selected to create an ‘ON’ situation. Buds were sampled at the terminal position of bourse shoot spurs, bearing no fruit on ‘OFF’ trees or from bourses carrying at least one fruit on ‘ON’ trees. In our conditions, full bloom occurred the 14th April 2010. Five harvests were performed between 10 and 12 a.m. from April to August 2010, at 15, 28, 48, 77 and 119 DAFB, corresponding to day 118, 131, 151, 180 and 222 in the following manuscript. This period was considered to cover floral induction, initiation and the early beginning of flower differentiation in spur apical buds [29]. Four trees per treatment and three buds per tree were sampled at each harvest date (Additional file 1: Figure S1). SAM were dissected from the terminal buds and immediately frozen in liquid nitrogen. Three trees and three buds over five time points were used for mRNA profiling by qRT-PCR. Based on mRNA profiling by qRT-PCR of key flowering genes, three dates among the five were chosen. On each of these dates, three buds from the four sampled trees were used for microarray analyses (Additional file 1: Figure S1).

In the spring following the collection of buds (2011), the proportion of flowering terminal buds was observed. Return to bloom was characterized on lateral branches depending on the treatment applied, i.e*.* ‘ON’ and ‘OFF’ trees. Trees conventionally thinned in 2010 were used as controls to evaluate the effect of the deflowering versus no-thinning treatments. Five trees per treatment were observed, evaluating 132 to 344 terminal buds per tree.

### RNA extraction and quantification

Total RNA was extracted from individual apical buds using the NucleoSpin® RNA II’ RNA kit (Macherey-Nagel GmbH & Co. KG, Düren, Germany), including DNA digestion on a column, according to the manufacturer’s instructions. Quantification of total RNA was performed using Infinite® 200 PRO NanoQuant (Tecan Trading AG, Männedorf, Switzerland) and RNA quality was controlled using an Agilent 2100 Bioanalyzer (RNA integrity number > 7) (Agilent Technologies, Santa Clara, USA).

### RNA profiling by qRT-PCR

Three key flowering genes that had been previously characterized in apple were used as molecular markers for flowering transition for the qRT-PCR study (*MdTFL1*, *MdAP1a* and *MdAP1b*) [50, 53]. Along with *MdFT1*, *MdFT2*, *AFL1* and *AFL2*, these genes enabled the comparison of our results with previous studies [36, 50, 53] and the identification of the critical period for FI in our experiment.

RNA transcript level was quantified by Quantitative Reverse Transcription PCR (qRT-PCR) following the advice of Udvardi *et al.* [104]. Using the same samples as for the microarray analyses, three trees and three buds per tree over five time points were used for mRNA profiling by qRT-PCR, except for day 118 and 131 where only two trees were available. First-strand cDNA was synthesized from 500 ng of total RNA using oligo (dT) 18 primers and the reverse transcriptase SuperScriptIII (Invitrogen, Carlsbad, CA, USA) according to the manufacturer’s instructions. Quality of cDNA was assessed using two pairs of primers 700 bp apart and located at the 5′ and 3′-end of the reference gene *ELONGATION FACTOR-1α* (*EF-1α*). Samples which threshold cycle (Ct) difference between the 5′ and 3′-end exceeded three cycles were not used further in the analysis.

Presence of genomic DNA contamination was assessed by designing primers for PCR either on the junction of two exons to amplify only cDNA or on separated exons to amplify longer fragments on genomic DNA (Additional file 2: Table S1). Gene sequences used for the primer design were retrieved from the Genome Database for Rosaceae (GDR, http://www.rosaceae.org/) Malus x domestica Whole Genome v1.0 Assembly & Annotation.

For the qRT-PCR reactions, 2 μl of the cDNA samples (diluted 1:20) was used as template in a 6-μl final reaction volume containing 3 μl of 2X Sybr green® (Roche) and 3 μM of each primer. Real-time PCR reactions were run on the LightCycler® 480 (Roche) with an initial denaturation of 5 min at 95 °C followed by 50 cycles of 10 s at 95 °C, 30 s at 55 °C and 15 s at 72 °C. The PCR products were analyzed by melting curve analysis to verify the presence of a gene-specific PCR product and the absence of genomic DNA contamination. The melting curve analysis was performed immediately after the PCR amplification using a single step at 95 °C for 1 min, 40 °C for 1 min and an annealing procedure starting at 65 °C to reach 95 °C with a time increment of 0.02 °C/s. Each reaction included negative and positive controls and each cDNA sample was analyzed using two technical replicates. Reactions showing difference in Ct higher than one cycle between the two technical replicates were not considered in the analysis. The PCR efficiencies were determined with a dilution range of a pool of all cDNA samples that was composed of seven data points (from 1:10 to 1:500). PCRs were run with two reference genes that are known to be stably expressed under the range of experimental conditions tested and have been previously used in apple: *HistoneH3* and *α-ACTIN* [22, 36, 51, 79]. Transcript levels were calculated with the LightCycler® 480 software (Version 1.5.0.39, Roche).

Statistical analyses of qRT-PCR results were performed using R [78]. Normalized transcript levels were analyzed by ANOVA type III, considering the treatment (‘ON’ v. ‘OFF’), date, tree, and bud nested in tree effects. The Student’s *t*-test was performed to estimate the significance of the difference between ‘ON’ and ‘OFF’ treatments at each date.

### Microarray analysis

The AryANE_v1 microarray employed consists of 126,022 probes corresponding to 63,011 probes each replicated in the forward and reverse sense and including all the predicted genes from the ‘Golden Delicious’ apple genome sequence and controls [15]. Probe length was 60 bp and probes were combined in a Nimblegen microarray (Roche Nimblegen, Madison, USA).

All spot comparisons were made between ‘ON’ (control) and ‘OFF’ trees at day 131, 151 and 222 (28, 48 and 119 DAFB). These time points were chosen to represent floral induction, floral bud initiation and the first steps of flower differentiation respectively for spur apical buds [29]. For each time point, total RNA of two trees per treatment and three buds per tree and per date, corresponding to six separate RNA extractions mixed in equal quantity, constituted a biological replicate (Additional file 1: Figure S1). Two independent biological replicates were performed with the ‘ON’ and ‘OFF’ cDNA clones labeled with Cy3 and Cy5 fluorescent dyes, respectively. A dye-swap was performed to eliminate any bias resulting from the two fluorescent dyes.

Labeling of microarray samples, microarray hybridization, scanning, quantification, normalization (with the lowess method) and analysis were performed as described in Celton et al. [15].

### Gene filtering and functional categorization

Identification of transcripts that were significantly differentially expressed was performed using R [78] as detailed in the procedure R available on GitHub (https://github.com/Baptiste-Guitton/Microarray_biennial_bearing_apple). Only anti-sense probes, that hybridize sense transcripts, were considered in the analysis. Transcripts with log difference higher than 1 or lower than −1 associated with a significant p-value (<0.01) and probe specificity lower or equal to 2 were selected for further analysis. Genes were then grouped depending on their expression profile. At each sampling date, each gene was tagged as “a” if its log^2^ ratio was less than −1, “b” if its log^2^ ratio was more than 1 or “c” if it exhibited no differential expression. The 3^3^ possible combinations of a, b and c (minus 1 because the genes not differentially expressed at the three dates were not considered) constituted 26 possible groups of genes. Construction of Venn diagrams was performed using the R function “draw.triple.venn” (library VennDiagram). Functional categorization analyses were performed using *Arabidopsis thaliana* genes homologous to apple transcripts present in the microarray, based on BLAST analysis against the TAIR database (https://www.arabidopsis.org/Blast/). Functional classification of genes was made using the web tool agriGO [25]. First, Gene ontology (GO) was investigated for the whole subset of genes and then detailed date by date. Secondly, to identify enriched GO terms in the lists of differentially expressed genes at specific dates of the microarray analysis, singular enrichment analyses (SEA) were performed [25]. Searches for protein interactions within gene sets were undertaken using STRING v9.1 [30].

### Genomic position of genes and QTL

The genomic position of genes that showed significant differential expression for at least one of the three harvest dates was retrieved using the best hit reports of blastp of the *Malus* x *domestica* genome v1.0 proteins file (Malus_x_domestica.v1.0_gene_pep_function_101210.formated.xls) made available by the Genome Database for Rosaceae (GDR, http://www.rosaceae.org/).

Previous studies based on a QTL detection approach in an apple segregating population, ‘Starkrimson’ x ‘Granny Smith’ (STK x GS), identified eight genomic regions involved in control of biennial bearing and inflorescence yield [24, 33]. Positions of SSR markers used to detect QTL were retrieved either by blasting the marker sequence (primer or amplicon) available on the Hidras database (Hidras, November 2014, http://www.hidras.unimi.it/index.php) to the apple reference genome [106], or by using the Genome Database for Rosaceae (GDR, http://www.rosaceae.org/, file Malus_x_domestica.v1.0.markers.xls). For SNP markers, the position of the gene prediction (MDP) used to design the marker [33] was considered as the genomic position of the marker (Additional file 2: Table S2). QTL were defined between the two genetic markers flanking the QTL on the STK x GS integrated genetic map (Additional file 2: Table S3). Since the chr10 QTL was detected at the very bottom of the linkage group and flanking markers exhibited inverted order between the genetic and the physical maps, in our analyses we considered the lower part of chr10, below the MS06g03 genetic marker, as the QTL confidence interval.

## Results

### Evaluation of trees’ return to bloom

**Fig. 1:**
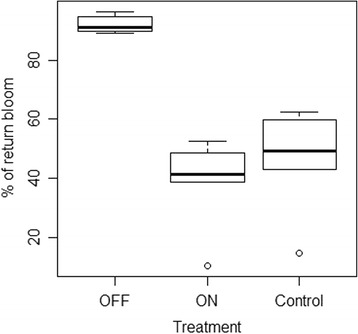
Proportions of shoot apical buds of ‘Gala’ apple trees that flowered in the year after a deflowering treatment (‘OFF’, bearing no fruit), fruiting trees (‘ON’, bearing heavy crop) and control trees (bearing a commercial crop)

Hand-thinned trees treated as ‘OFF’ in 2010 massively induced flowers, with 92.3 % of their buds being floral in 2011 whereas 62.8 % of the buds of ‘ON’ trees remained vegetative (Table 1, Fig. 1). However, 38.2 % of the buds of ‘ON’ trees developed flowers in 2011, this percentage being on average lower than for the conventional thinned control trees (45.8 %). In ‘ON’ and control trees, variation among trees was higher than among ‘OFF’ trees, characterized by a higher standard deviation (Table 1).

**Table 1:** Characterization of flowering intensity in 2011 of apple trees bearing no crop (‘OFF’), heavy crop (‘ON’) or conventional crop (‘Control’) in 2010. ‘ON’ and ‘OFF’ trees labeled 3, 4 and 5 were used for mRNA relative quantification by qRT-PCR, and trees labeled 2 to 5 were used for mRNA microarray analyses

Tree	Treatment	Number of meristem evaluated	Percentage of flowering meristems in 2011	Mean	Standard deviation
1	OFF	206	89.81 %	92.3 %	0.032
2	OFF	304	91.12 %		
3	OFF	289	89.27 %		
4	OFF	285	96.49 %		
5	OFF	132	94.70 %		
1	ON	217	38.71 %	38.2 %	0.166
2	ON	280	52.50 %		
3	ON	293	10.24 %		
4	ON	298	41.28 %		
5	ON	338	48.52 %		
1	Control	344	14.53 %	45.8 %	0.192
2	Control	207	59.90 %		
3	Control	223	43.05 %		
4	Control	219	49.32 %		
5	Control	319	62.38 %

### Screening for expression of flowering genes by qRT-PCR and in the microarray

The expression profile of transcript levels of key flowering genes previously published in various apple cultivars were studied by qRT-PCR over five time points. This enabled us to compare our results with the literature, interpret the profiles based on the known function of these genes, and to choose three out of five time points available for microarray analyses.

**Fig. 2:**
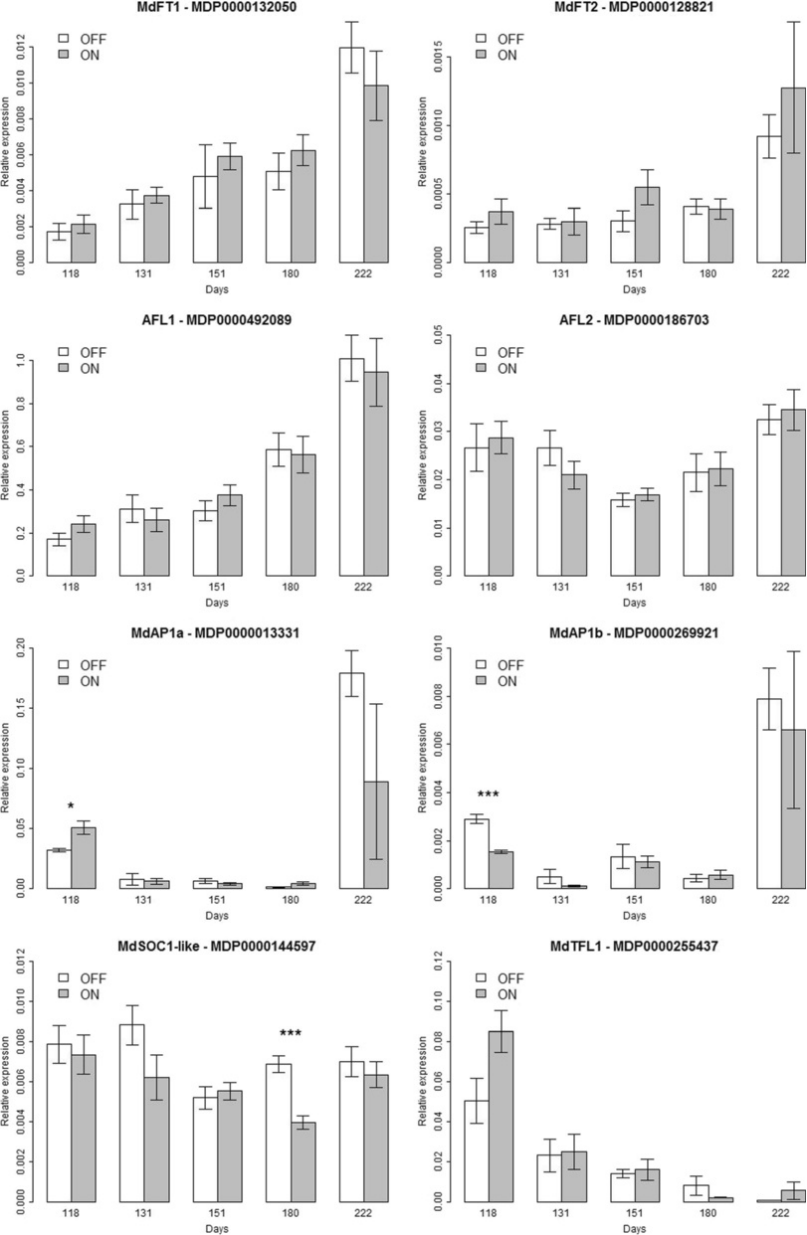
Expression pattern of *MdFT1*, *MdFT2*, *AFL1*, *AFL2*, *MdAP1a*, *MdAP1b*, *MdSOC1-like* and *MdTFL1* during the growing season in ‘Gala’ apple trees measured by quantitative real-time PCR using spur apical buds harvested at day 118, 131, 151, 180 and 222. Graphics represent the average of the nine data points per date and per treatment with associated standard deviation. Significance of the difference of relative expression between ‘ON’ and ‘OFF’ trees for each studied gene and at each time point estimated by Student *t*-test was reported on the graphics (*, *p*-value < 0.05; **, *p*-value < 0.01; ***, *p*-value < 0.001). Relative expression was calculated using the *HistoneH3* housekeeping gene

Specific and unique PCR-products were amplified for *MdFT1*, *MdFT2*, *AFL1*, *AFL2*, *MdAP1a*, *MdAP1b*, *MdSOC1-like* and *MdTFL1*, which was confirmed by melting curve analyses and by sequencing of PCR amplicons (data not shown). Transcript levels were normalized against the mRNA expression level of the reference gene *HistoneH3* of apple, which enabled us to compare our results with previous studies [51] (Fig. 2); and similar profiles were confirmed using the *α-ACTIN* reference gene (Additional file 3: Figure S2). Transcript levels of seven of these genes, *AFL1* and *AFL2*, *MdAP1a* and *MdAP1b*, *MdFT1* and *MdFT2*, and *MdTFL1*, were used for microarray validation and revealed similar expression patterns with strong correlations (Additional file 4: Figure S3).

Flowering genes such as *MdFT1*, *MdFT2* and *AFL1* showed increasing transcript levels during the growing season but no significant differences in the expression pattern were observed between ‘ON’ and ‘OFF’ trees (Fig. 2, Additional file 2: Table S4). The relative expression profiles of *MdSOC1-like* (MDP0000144597) and *AFL2* (MDP0000186703) were similar, decreasing from day 118 to 151 and then slightly increasing up to day 222. However, *MdSOC1-like* transcript levels of ‘OFF’ trees were above this tendency at day 131 and 180. The difference between ‘ON’ and ‘OFF’ trees was only significant at day 180.

*MdAP1a* and *MdAP1b* exhibited similar expression patterns (Fig. 2), as they were expressed at low level in the buds until day 180 and then significantly increased at day 222. At this date, ‘OFF’ trees showed higher levels of transcript for both *AP1* genes, the difference between the two treatments was not significant because of high standard error observed in ‘ON’ trees, (Additional file 2: Table S5). *MdTFL1* (MDP0000255437) had a contrasting profile as its expression decreased rapidly from day 118 and was very low at day 180. Transcript levels were higher in ‘ON’ trees than in ‘OFF’ trees at day 118, the difference was just below the significance threshold (*p*-value = 0.054) (Additional file 2: Table S5).

Finally, as the *FT*/*TFL1* gene family is the main known flowering gene family in apple, we paid particular attention to these genes in the microarray. In the microarray experiment, both *MdFT1* (MDP0000132050) and *MOTHER OF FT* (*MdMFTa* - MDP0000449224) expression levels increased between day 131, 151 and 222; however, no differential expression was observed between treatments. Both *TFL1* copies (*MdTFL1* - MDP0000126761 and *MdTFL1a* -MDP0000255437) exhibited the expected expression profile, with expression decreasing from day 131 to 222, but without differential expression. Neither *MdFT2* (MDP0000144597), *BROTHER of FT* (MDP0000812208 and MDP0000867916) nor *TFL2* (*MdLHP1a*, MDP0000293596 and *MdLHP1b*, MDP0000240165) showed significant differential expression in the microarray between treatments at any time point.

Also, three apple homologous copies of *SOC1* (MDP0000314765, MDP0000060753 and MDP0000144597) were not differentially expressed, their expression remained stable and constant (around log = 5) over time points. Neither *CENTRORADIALIS* (*MdCENa*, MDP0000761080 and *MdCENb*, MDP0000127457) nor *MOTHER OF FT* (*MdMFTb*, MDP0000208806) was expressed in the apical buds at any dates.

### Overview of genes differentially expressed in the microarray

Among the 63,011 probes targeting sense transcripts, only transcripts highly specific to the targeted gene (46,261 probes) with log2 intensity higher than log = 1 above the background for at least one sample (45,622 probes) were considered as positive for their expression. After the filtering procedure, a total of 648 transcripts were identified as significantly differentially expressed for at least one of the three time points, representing 1.03 % of the sense genes targeted on the microarray. In the following, we reported mainly to genes up or down-regulated in ‘ON’ trees, and mentioned the corresponding down and up-regulation in ‘OFF’ trees only when this was meaningful for gene interpretation.

**Fig. 3:**
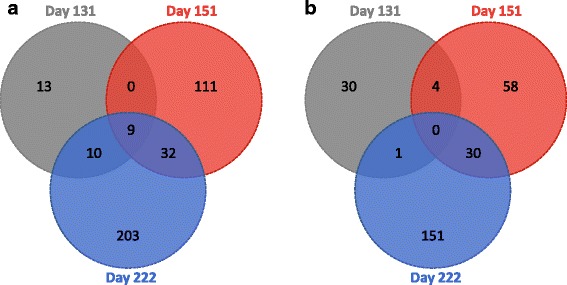
Generalized Venn diagram with three sets of transcripts at day 131 (grey), 151 (red), and 222 (blue) and their intersections for up-regulated genes between ‘ON’ (control) and ‘OFF’ ‘Gala’ apple trees in (**a**) ‘ON’ trees (bearing heavy crop that inhibits flower formation) and (**b**) ‘OFF’ trees (bearing no crop and initiating flower formation)

**Fig. 4:**
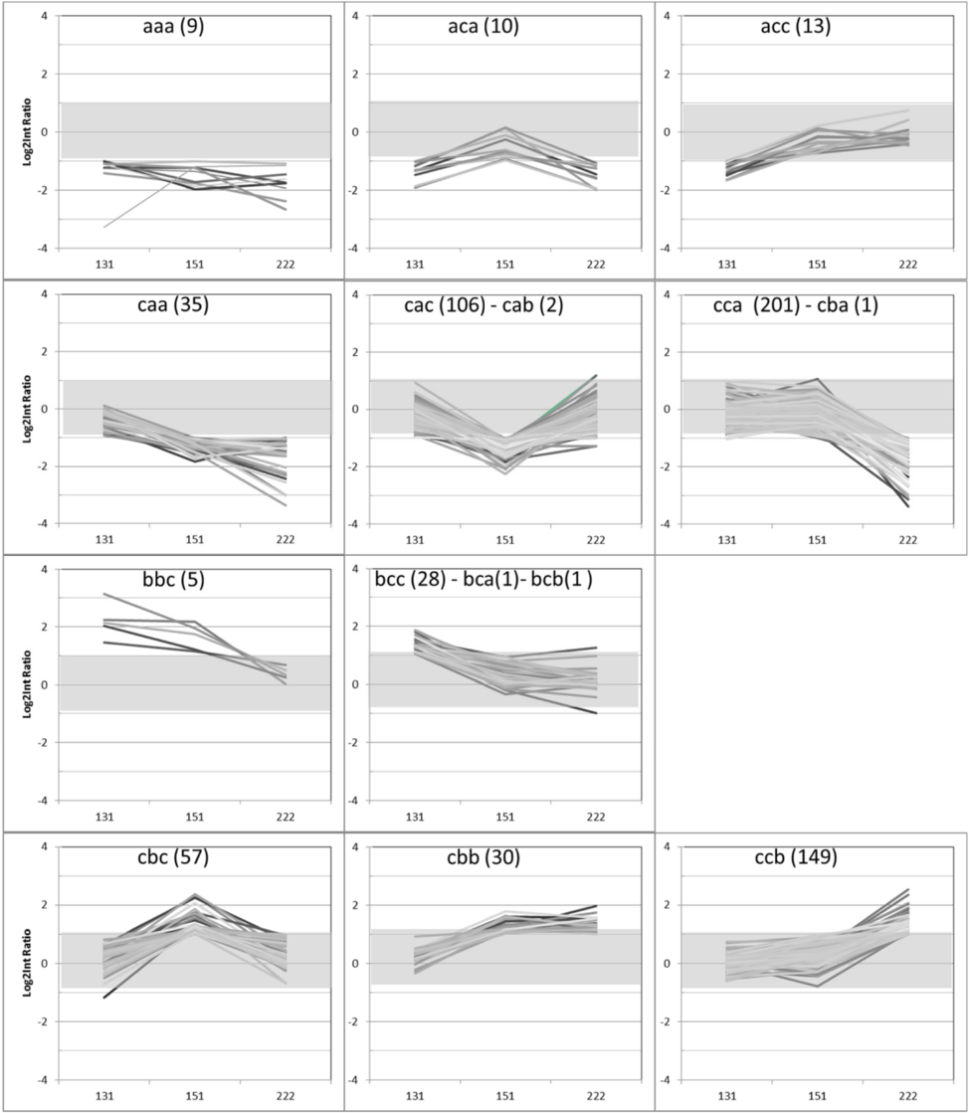
Gene expression profiles visualized in a user-defined number of 11 groups. At each sampling date, each gene was tagged as “a” if its log^2^ ratio was less than −1, “b” if its log^2^ ratio was more than 1 or “c” if it exhibited no differential expression. Groups are numbered 1–11 (by rows and from left to right), and the total number of genes per set is noted into parentheses. For each diagram, the x-axis shows the three time points (day 131, 151, and 222), while the y-axis corresponds to fold change in the set of genes. Negative log^2^ ratio corresponds to transcripts up-regulated in trees inhibiting flowering (‘ON’), and positive log^2^ ratio corresponds to transcripts up-regulated in trees initiating flowering (‘OFF’). The grey domain indicates the −1 and +1 limits used to filter gene expression

The number of up-regulated (ratio < −1) and down-regulated (ratio > 1) transcripts in ‘ON’ trees increased through time, with a number of up-regulated transcripts higher than of down-regulated transcripts (Fig. 3). Among these transcripts, 32 were up-regulated in ‘ON’ trees at day 131, whereas 35 were down-regulated trees. At day 151, 152 and 92 transcripts were up-regulated and down-regulated in ‘ON’ trees, respectively. At day 222, 254 transcripts were up-regulated in ‘ON’ trees and 182 down-regulated. Nine transcripts were consistently up-regulated in ‘ON’ trees over the three dates, 19 transcripts were common to day 131 and 151, 41 transcripts to day 151 and 222 and 19 transcripts to day 131 and 222. Only four transcripts were down-regulated in ‘ON’ trees at both day 131 and 151, 30 were common between day 151 and 222, and one transcript was in common to day 131 and 222. No genes in common were down-regulated during the time course of the experiment in ‘ON’ trees. Differential transcript profiles in apple buds from day 131 to 222 were visualized and transcripts that shared similar differential expression profiles were grouped into a user-defined number of 11 groups (Fig. 4). Among the 26 possible profiles described above, only 15 were observed. Four groups contained 1 or 2 genes only and were therefore gathered with another group of similar profile (Additional file 2: Table S6). Groups 1–6 contained transcripts up-regulated in apical buds of ‘ON’ trees, whereas clusters 7–11 contained transcripts down-regulated.

Of the 648 transcripts differentially expressed in our experiment, 535 were homologous to an *Arabidopsis* gene with a significant E-value, while the 113 remaining transcripts did not share significant homology with any *Arabidopsis* gene. The 535 annotated apple transcripts corresponded to 426 unique *Arabidopsis* genes.

Single Enrichment Analysis (SEA) using AgriGO showed that the ‘response to stimulus’ GO (*p*-value = 7.3E-16) was the most significantly over represented in the subset of genes differentially expressed for at least one of the three time points. At day 131, the genes with sequence homology to response to stimulus GO including the response to abiotic stress, the response to stress and the cellular response to stimulus GO were significantly up-regulated (Additional file 5: Figure S4A, Additional file 2: Table S7A). At day 151, four main biological processes were differentially expressed: (i) the response to stimulus, with the response to stress GO being the most significant of the dataset (*p*-value = 8.90E-11) with, among others, the response to oxidative stress GO (*p*-value = 8.40E-05); (ii) GO belonging to cellular processes; (iii) cell wall biogenesis (*p*-value = 1.50E-05) as part of the cellular component biogenesis GO; and (iv) metabolic processes, including the cellular carbohydrate biosynthetic process (*p*-value = 0.00094) and the lipid metabolic process (*p*-value = 0.00021) (Additional file 5: Figure S4B, Additional file 2: Table S7B). At day 222, the SEA analysis showed that the response to stimulus was still the most significantly over-represented GO (p-value = 1.50E-10), which included the response to gibberellin stimulus (p-value = 4.30E-5), the response to jasmonic acid stimulus (*p*-value = 5.60E-5) and the response to oxidative stress (*p*-value = 2.60E-06). In the developmental process GO, which ends in anatomical structure development (*p*-value = 0.0037), 35 transcripts were found to be differentially modulated between the two treatments. The metabolic process GO included the carbohydrate biosynthetic process (*p*-value = 0.0015) and the transcription GO (*p*-value = 8.40E-05) in which 31 transcripts were differentially expressed between the treatments (Additional file 5: Figure S4C, Additional file 2: Table S7C).

#### GDE in metabolic processes suggest that apical buds of trees bearing a heavy crop are starved

**Fig. 5:**
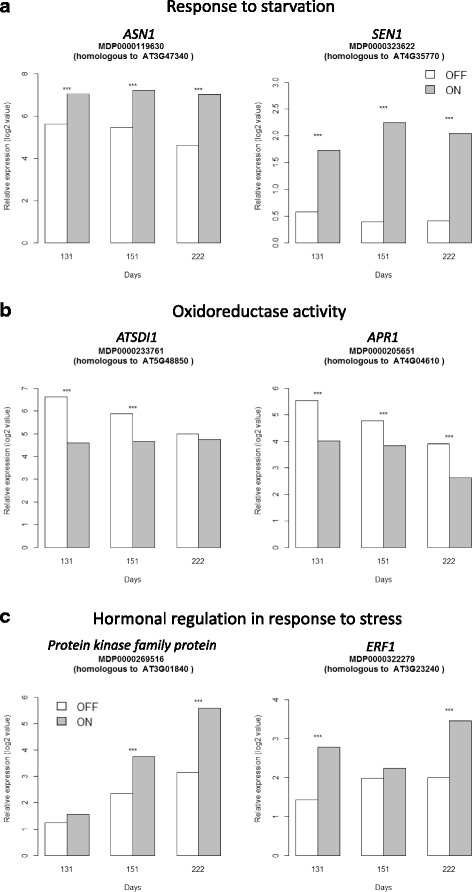
Kinetics of the relative expression values (log^2^ ratio) of transcripts differentially expressed involved in response to starvation (**a**), oxidoreductase activity (**b**) and hormonal regulation in response to stress (**c**) in apple spur apical buds between trees initiating flowering (‘OFF’) and trees inhibiting flowering (‘ON’) at three developmental stages (day 131, 151 and 222). The array data were normalized with the lowess method. Normalized intensities (i.e. expression levels) were then subtracted from the background. Stars (***) indicate significant differences of expression between the two treatments

Among the nine transcripts up-regulated in ‘ON’ trees over the three dates, five are homologous to genes involved in the response to stresses: *ASN1* (*GLUTAMINE-DEPENDENT ASPARAGINE SYNTHASE 1*; MDP0000119630 and MDP0000096208), involved in cellular response to sucrose starvation, *SEN1* (*SENESCENCE 1*; MDP0000323622 and MDP0000121259), a senescence-associated gene that is strongly induced by phosphate starvation, and MDP0000223224, a putative sugar phosphate exchanger (Fig. 5a, Table 2, Additional file 2: Table S8A).

**Table 2:**
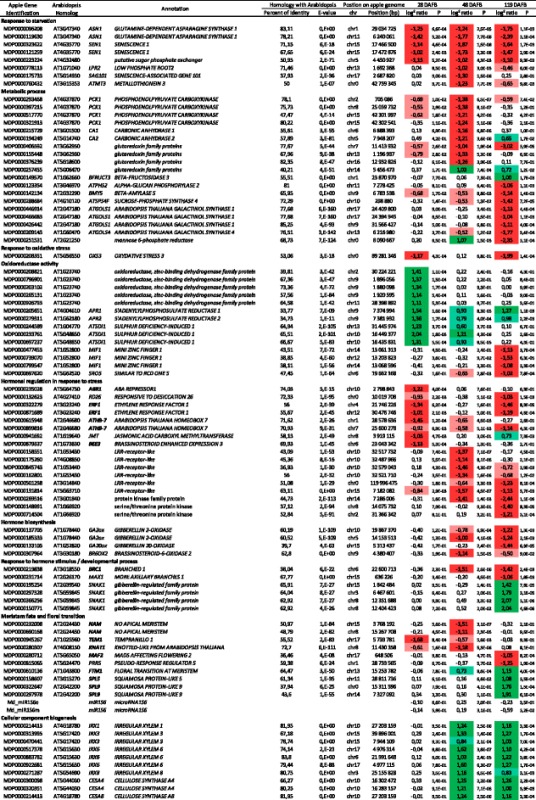
Selection of differentially expressed transcripts in apple spur apical buds between ‘ON’ and ‘OFF’ trees. The gene identifier is reported along with the *Arabidopsis* gene homolog, a short annotation, BLAST results against TAIR database (percentage of identity and E-value), position on the apple genome (chromosome and position in bp), and the pattern of expression at day 131, 151 and 222. Negative log^2^ ratios correspond to transcripts up-regulated in ‘ON’ trees

Significant differences (p-value ≤ 0.05) between the two treatments were colored. Light green indicate log² ratio values lower than 1 and dark green indicate values higher than 1. Light red indicate log² ratio values higher than -1 and dark red indicate values lower than -1.

One transcript homologous to *LPR2* (*LOW PHOSPHATE ROOT2*; MDP0000778113) involved in cellular response to phosphate starvation was found to be over-expressed in ‘ON’ trees at day 151. At this same date, primary carbohydrate metabolism in apical buds of ‘ON’ trees was oriented towards gluconeogenesis as indicated by the up-regulation of four homologous copies of *PCK1* (*PHOSPHOENOLPYRUVATE CARBOXYKINASE*; MDP0000293468, MDP0000321913, MDP0000397215 and MDP0000517770). Two homologous transcripts involved in carbon utilization *CA1* and *CA2* (*CARBONIC ANHYDRASE*; MDP0000215729 and MDP0000194249), and four glutaredoxin family proteins homologs (MDP0000376239, MDP0000155448, MDP0000406592, and MDP0000257455) involved in the cellular homosteasis GO were also up-regulated in ‘ON’ trees. We also noticed a transcript homologous to *ATMT3* (*METALLOTHIONEIN 3*; MDP0000760432) participating in cellular copper ion homeostasis, known to be involved in the response to fructose stimulus, growth and in the positive regulation of the flavonoid biosynthetic process as well as a transcript homologous to *SAG101* (*SENESCENCE-ASSOCIATED GENE 101*; MDP0000175733), which encodes an acyl hydrolase involved in senescence, even though both these transcripts had relatively low percentage of homology, 50 % and 38 %, respectively (Table 2, Additional file 2: Table S8B).

Among transcripts that were up-regulated in ‘ON’ trees at day 222, we particularly note MDP0000142134, a homolog of *BMY5* coding for a beta-amylase, and MDP0000123354, a homolog of *ATPHS2* coding for an alpha-glucan phosphorylase, both involved in starch degradation. At the same time, transcripts coding for a beta-fructosidase *BFRUCT3* homolog (MDP0000149570) and sucrose-phosphate synthase *ATSPS4F* (MDP0000288684) were down and up-regulated, respectively. In addition, galactinol synthase homologs involved in the carbohydrate biosynthetic process (MDP0000426442, MDP0000446914, MDP0000466683 and MDP0000209143) and a mannose 6-phosphate reductase homolog (MDP0000251531) were up-regulated in ‘ON’ trees (Table 2, Additional file 2: Table S8C).

#### Apical buds of trees bearing a heavy crop are in unfavorable cellular redox status

**Fig. 6:**
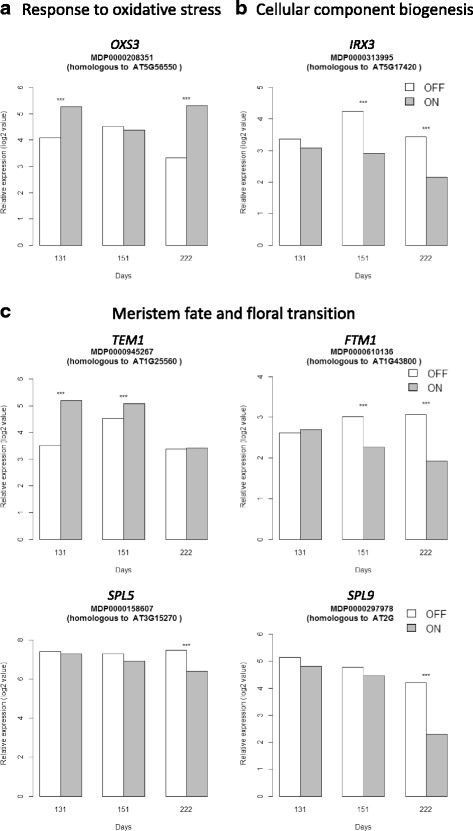
Kinetics of the relative expression values (log^2^ ratio) of transcripts differentially expressed involved in response to oxidative stress (**a**), cellular components biogenesis (**b**) and meristem fate and floral transition (**c**) in apple spur apical buds between trees initiating flowering (‘OFF’) and trees inhibiting flowering (‘ON’) at three developmental stages (day 131, 151 and 222). The array data were normalized with the lowess method. Normalized intensities (i.e. expression levels) were then subtracted from the background. Stars (***) indicate significant differences of expression between the two treatments

The oxidoreductase molecular function was significantly over-represented, with eight, 21 and 25 genes up-regulated at day 131, 151 and 222, respectively. This indicates that the reduction of oxygenized molecules was active over the three time points of the experiment. One transcript particularly relevant to the cellular redox status, homologous to *OXS3* (*OXYDATIVE STRESS 3*; MDP0000208351), which is involved in the tolerance to oxidative stress (Table 2), was up-regulated in ‘ON’ trees at day 131 and 222 (Fig. 6a).

Transcripts involved in the oxidoreductase activity and the catalytic activity GO, which were both significantly over-represented, were down-regulated at day 131 in apical buds of ‘ON’ trees (Additional file 2: Table S7A and S8A). Five homologous copies of AT1G23740, a quinone oxidoreductase-like protein, possibly involved in detoxification of reactive carbonyls, were found in the transcript set. Genes belonging to the sulfate reduction and assimilation GO were also down-regulated in ‘ON’ trees. Organic sulfur compounds, such as thiols, sulfolipids, glucosinolates or alliins, are known to play important roles in the normal plant lifecycle and in protection against stress and pathogens. Homologs of *APR1* and *APR2* (*5′ADENYLYLPHOSPHOSULFATE REDUCTASE*; MDP0000205651 and MDP0000279311) which encode key enzymes of the assimilatory sulfate reduction pathway and for which a decrease in enzyme activity leads to sulfate accumulation in the plant, were down-regulated in ‘ON’ trees for the three time points (Fig. 5b). However, the difference was only statistically significant at day 131 for the homolog of *APR2* and at day 131 and 222 for *APR1* homolog. Two homologous copies of *ATSDI1* (*SULFUR DEFICIENCY-INDUCED 1*; MDP0000233761 and MDP0000697237), which has a role in the regulation of stored sulfate pools and is induced by sulfur starvation, were down-regulated in ‘ON’ trees at both day 131 and 151 (Fig. 5b). Another homologous copy of *ATSDI1* (MDP0000244589) was down-regulated in ‘ON’ trees at day 131 only. Finally, three transcripts homologous to *MIF1* (*MINI ZINC FINGER 1*, MDP0000477453, MDP0000739070 and MDP0000799547), an oxidoreductase involved in multiple hormonal regulation, were up-regulated at day 222 in apical buds of ‘ON’ trees as well as a transcript homologous to *SRO5* (*SIMILAR TO RCD ONE 5*; MDP0000697620), a gene involved in the oxygen and reactive oxygen species metabolic process, which was up-regulated at day 222 (Table 2).

#### GDE involved in hormonal regulation

At day 131, 13 transcripts homologous to genes involved in hormonal regulation in response to stress were up-regulated in apical buds of ‘ON’ trees, among them: *RD26* (*RESPONSIVE TO DESICCATION 26*; MDP0000132623), involved in response to abscisic acid stimulus; *ABR1* (*ABA REPRESSOR1*; MDP0000235028), a member of the *ETHYLENE RESPONSE FACTOR* (*ERF*) subfamily involved in regulation of ABA-mediated stress response; *ATHB-7* (*ARABIDOPSIS THALIANA HOMEOBOX 7*; MDP0000615948 and MDP0000899816), involved in abscisic acid-mediated signaling and response to abscisic acid stimulus. Four homologous transcripts were also involved in response to ethylene: *JMT* (*JASMONIC ACID CARBOXYL METHYLTRANSFERASE*; MDP0000941692), which catalyzes the formation of methyljasmonate from jasmonic acid; and *BEE3* (*BRASSINOSTEROID ENHANCED EXPRESSION 3*; MDP0000879337), a transcription factor induced by brassinosteroid, auxin and ethylene, and repressed by abscisic acid; *ERF1* (*ETHYLENE RESPONSE FACTOR 1*; MDP0000322279 and MDP0000871689) (Fig. 5c). Moreover, six Leucine-Rich-Repeat (LRR) receptor-like transcripts were up-regulated in ‘ON’ trees, in particular MDP0000158551, MDP0000162801, MDP0000175260 and MDP0000845743 at day 151, MDP0000501298 at day 222, and MDP0000131814 at day 151 and 222 (Table 2). These LRR proteins are known to be involved in protein amino acid phosphorylation, which is one of the most important and frequent regulation mechanisms in plants.

At day 151, two transcripts homologous to *GA2ox* (*GIBBERELLIN 2-OXIDASE*; MDP0000137705 and MDP0000185333) and one homolog of *GA20ox* (*GIBBERELLIN 20-OXIDASE*; MDP0000133105) were up-regulated in ‘ON’ trees. We also noted *BRC1* (*BRANCHED 1*; MDP0000219838), a transcription factor indirectly required for the auxin-induced control of apical dominance and known to arrest axillary bud development, prevent axillary bud outgrowth and delay early axillary bud development, along with *BR6OX2* (*BRASSINOSTEROID-6-OXIDASE 2*; MDP0000307964), which encodes a cytochrome P450 enzyme that catalyzes the last reaction in the production of brassinolides which regulate the plant growth and productivity, and are known to alleviate various biotic and abiotic stress effects.

*SNAK1* homologs coding for proteins involved in responses to gibberellin stimulus were down-regulated at day 222 (MDP0000195254, MDP0000297328, MDP0000366256 and MDP0000150771). In the developmental process GO, in addition to *BRC1* previously mentioned, another transcript homologous to a major gene involved in branching control was up-regulated in ‘ON’ trees, *MAX1* (*MORE AXILLARY BRANCHES 1*, MDP0000231714). This gene is a specific repressor of vegetative axillary buds generated by the axillary bud, known to be involved in the regulation of meristem organization, positive regulation of flavonoid biosynthetic process, carotenoid biosynthetic process, secondary shoot formation and auxin polar transport (Table 2).

#### Floral repressor transcripts are up-regulated in apical buds of trees bearing a heavy crop, whereas floral enhancer transcripts are up-regulated in defruited trees

Among transcripts involved in the flowering time pathway, MDP0000945267 which shares 55.5 % of homology with *TEM1* (*TEMPRANILLO 1* or *RAV2*) in Malus x domestica Whole Genome v1.0 was found significantly up-regulated in ‘ON’ trees at day 131 (Fig. 6c). *TEM1* is known as a floral repressor possibly linked to the photoperiod and GA-dependent flowering pathways. Also, two homologous copies of the transcription factor *NO APICAL MERISTEM* (*NAM*) (MDP0000690168 and MDP0000232008) were up-regulated in trees inhibiting floral induction (‘ON’ trees) at day 151. This transcription factor belongs to the NAC protein family which is involved in various plant developmental processes such as SAM development and stress inducible floral induction. The transcription factor *KNAT1* (*KNOTTED-LIKE FROM ARABIDOPSIS THALIANA*; MDP0000280307), known to be expressed in mid-meristem to promote leaf fate, was also up-regulated at day 151 in SAM of ‘ON’ trees.

Two other homologous transcripts of interest were up-regulated in ‘ON’ trees, *MAF2* (*MADS AFFECTING FLOWERING 2*, MDP0000280712 having 76.2 % of homology with *FLOWERING LOCUS C-Like* in Japanese pear) and *PRR5* (*PSEUDO-RESPONSE REGULATOR 5*, MDP0000815065), but at day 222 only. In Arabidopsis, *MAF2* is known to be homologous to *FLOWERING LOCUS C* and *M (FLC and FLM*), which are repressors of flowering and major determinants of natural flowering time variation in response to cold temperatures whereas *PRR5* encodes components involved in a negative feedback loop in the circadian clock in *Arabidopsis thaliana* and could be a floral inhibitor.

Furthermore, one homologous copy of *FTM1* (*FLORAL TRANSITION AT MERISTEM*, MDP0000610136) coding for a stearoyl-ACP desaturase was down-regulated in ‘ON’ trees at both day 151 and 222, and therefore up-regulated in ‘OFF’ trees (Fig. 6c). Three transcripts sharing homology with *SPL* (*SQUAMOSA PROTEIN-LIKE*) family members were also down-regulated in ‘ON’ trees at day 222 (or up-regulated in ‘OFF’ trees), *SPL5* (MDP0000158607) and *SPL9* (MDP0000297978 and MDP0000322647) (Fig. 6c). In Arabidopsis, these transcripts are known to be involved in the vegetative to reproductive phase transition and flowering regulation. Expression of both *SPL5* and *SPL9* is regulated by the microRNA *miR156*. However, in our experiment *miR156* did not showed differential expression between the two treatments (Table 2).

#### Apical buds of defruited trees are under active biogenesis at day 151 and 222

The SEA analysis indicated that cell wall biosynthesis GO, and metabolic processes including the cellular carbohydrate biosynthetic process and the lipid metabolic process were significantly up-regulated in ‘OFF’ trees (Additional file 2: Table S6B and S6C). Furthermore, processes linked to cellular processes and cellular component biogenesis ending in cell wall biogenesis were also up-regulated, consistently with an increased cellular activity and meristem re-organization activity in trees initiating flowering (‘OFF’ trees). Transcripts homologous to Arabidopsis members of the cellulose synthase family involved in secondary cell wall biosynthesis: *IRX1* (*IRREGULAR XYLEM 1*, MDP0000214413), *IRX3* (MDP0000313995 and MDP0000470441), *IRX6* (MDP0000517378, MDP0000883782 and MDP0000922681) and *IRX8* (MDP0000271287), along with the *CELLULOSE SYNTHASE A 4* (*CESA4*, MDP0000300098 and MDP0000320351) and *CESA8* (MDP0000214413) were up-regulated in ‘OFF’ trees at day 151 and 222, except *IRX8*, which was not significantly up-regulated at day 222 (Fig. 6b, Table 2, Additional file 2: Table S7B and S7C).

### Distribution of differentially expressed genes on the genome and co-localization with QTL for biennial bearing

Of the 648 differentially expressed genes in our experiment, 610 genes were positioned on one of the 17 chromosomes of the apple genome (Additional file 2: Table S9). These were evenly distributed over the 881.3 Mb of the genome, with on average one gene every 1.59 Mb (Additional file 6: Figure S5).

In total, 81 genes were located within the eight QTL regions linked to the control of biennial bearing in a ‘Starkrimson’ x ‘Granny Smith’ segregating population [33]. Of these 81 genes, 62 were annotated (Additional file 2: Table S10). The SEA analysis showed that the oxidoreductase activity was over-represented with eight genes (*p*-value 0.0012). Transcripts with sequence homology to seven leucine-rich repeat transmembrane protein kinases that were up-regulated in ‘ON’ trees were located within QTLs: MDP0000148991 (chr 8, day 222), MDP0000175260, MDP0000845743, MDP0000162801 and MDP0000158551 (chr 10, day 151), MDP0000269516 (chr 14, day 151 and 222), MDP0000131814 (chr 15, day 151 and 222).

Besides genes encoding a catalytic activity, genes involved in hormone stimulus response and plant developmental processes were located within QTLs. Within the chromosome 8 QTL interval, two copies homologous to a gene involved in the response to gibberellin stimulus (AT5G59845) were down-regulated in trees bearing heavy crop (‘ON’ trees) at day 222. On chromosome 14, the transcriptional regulator *SPL9* (MDP0000297978) involved in the vegetative to reproductive phase transition was down-regulated in ‘ON’ trees at day 222. In the QTL interval for inflorescence production (chr 15), one transcript homologous to the transcription factor *NO APICAL MERISTEM* (*NAM*) (MDP0000232008) was up-regulated in ‘ON’ trees at day 151.

## Discussion

### The defruiting treatment significantly induced flowers in apical buds of ‘OFF’ trees

The deflowering treatment that was applied to the trees proved effective in raising the amount of FI in the following spring. Trees induced to the ‘OFF’ state by removing all flowers at full bloom flowered heavily in the subsequent year, with more than 90 % of their buds producing inflorescences, with a low variance among treated trees. In contrast, heavy fruit load (‘ON’ trees) had a lower FI than the conventionally thinned trees used as controls. However, a proportion of buds (38.2 %) were induced to flower in the ‘ON’ trees, probably because of the tendency of ‘Gala’ to bear regularly [38]. It is likely that cultivars with a more severe biennial bearing tendency than ‘Gala’ might bear less after an ‘ON’ year. However, cultivars even prone to be regular can be pushed to irregularity by manipulations as shown by 62.8 % of the buds of ‘ON’ not-thinned trees that remained vegetative. Moreover, the high variability observed in ‘ON’ and control trees demonstrates that bud fate is heterogeneous and desynchronized within a tree, contrasting to the high degree of synchronism among apical buds observed in ‘OFF’ trees where flowers had been removed in the preceding year. The tendency to SAM synchronization versus desynchronization has been proposed to be genotype dependent [24, 57].

### Expression pattern of flowering genes as molecular markers of transition in apical buds

Flowering gene expression was studied using qRT-PCR during the critical phases of bud fate, from day 118–222, this period being assumed to include FI, transition to inflorescence meristem and early beginning of floral differentiation [29]. Because there is no single and obvious marker of FI in SAM of apple, we used qRT-PCR to study the expression patterns of key flowering genes. Our results are consistent with those of previous studies, especially for *MdFT1*, *MdSOC1-like*, *AFL1* and *AFL2* which exhibited similar expression patterns, although with lower levels of expression and less contrast between dates than those observed by Hättasch et al*.* [36]. In particular, we did not observe expression peaks of *MdTFL1* at day 180. Expression profiles of *MdTFL1*, *MdFT1* and *MdFT2*, and *MdAP1* were similar to those observed by Kotoda et al*.* [50], but again with less contrast between dates. The differences observed among the studies might be due to different normalization of the transcript level (in relation to that in the plants raised *in vitro* by [36]), or to the different environments in which the trees were grown and to the use of different cultivars.

*AP1* has been proposed as a molecular marker for flowering differentiation in Eucalyptus (Jaya et al. [43]). In Arabidopsis, the mode of action of *AP1* is dynamic, since it acts predominantly as a transcriptional repressor during the earliest stages of flower development; however, it activates regulatory genes required for floral organ formation at more advanced stages [48]. *AP1*’s role in floral organ formation has also been confirmed in apple [53]. In our qRT-PCR, the high expression of *MdAP1a* at day 118 in ‘ON’ trees might be interpreted as being related to its transcriptional repressor activity on FI, whereas the high expression levels of both *MdPA1a* and *MdAP1b* in the same trees after day 180 suggest that floral differentiation occurred after this date. Similarly, the lower expression of *MdAP1a* and *MdAP1b* at day 222 in ‘ON’ buds compared with that in ‘OFF’ buds may reflect the lower rate of FI in these buds. The lack of significant differential expression of *MdAP1* in the microarray may result from a mix of flowering and not-flowering buds and to the relatively early date considering the timing of flower differentiation that is assumed to occur between day 200 and day 240 [29]. The concomitant down-regulation of *MdTFL1* from day 180 is also consistent with previous studies showing that in apple, as in Arabidopsis, *TFL1* represses *AP1* expression [50]. The late expression of *MdPI*, enhanced by *MdAP1*, has been related to the development of floral organs in apple [98], which is consistent with the very low expression of *MdPI* detected at day 222 only (data not shown). This also suggests that the formation of floral whorls began around day 222 and that *MdPI* expression increases later in the season. The qRT-PCR profiles developed for key flowering genes, together with previous studies that have determined the timing of FI in apple [29, 35, 53], led us to choose three time points for the microarray analysis to represent the period of FI (day 131), floral bud initiation (day 151) and the beginning of flower differentiation (day 222). These three time points are the most likely to pinpoint some important mechanisms of biennial bearing and highlight differences between ‘ON’ and ‘OFF’ trees.

Moreover, the transcript levels of *AFL1* and *AFL2*, *MdAP1a* and *MdAP1b*, *MdFT1* and *MdFT2*, and *MdTFL1*, were highly correlated between qRT-PCR and microarray, and were considered as a validation of the microarray results.

### Triggering FI in apical buds of adult apple trees: a number of candidate processes

The results of the microarray analysis suggest that a number of processes could be involved in FI in apical buds of adult apple trees. Indeed, positioning the major genes differentially expressed in buds sampled in ‘ON’ or ‘OFF’ trees in the pathways described in the literature as controlling FI in model plants suggest that several of these pathways could be involved in FI triggering in adult apple trees. Many authors have previously underlined the multifactorial nature of FI in plants, with accelerated flowering in response to shade, drought, low nutrients, decreased light quality, heat and general oxidative stress [34, 59, 64, 66]. In perennial tropical trees, in absence of cold temperatures, FI can be triggered by water stress or other stressful practices [16, 90, 93].

### Starvation and unfavorable redox status in apical buds of trees bearing heavy crop

**Fig. 7:**
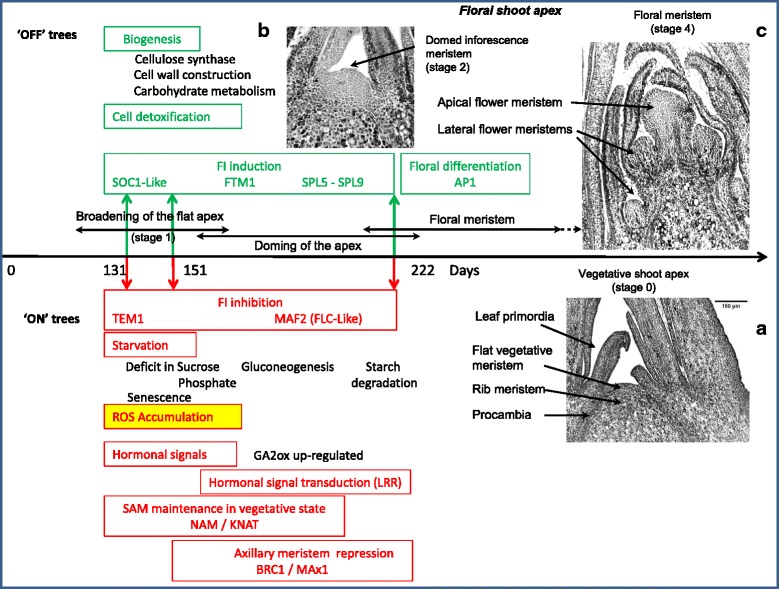
Main metabolic, hormonal and redox status regulations revealed by microarray analyses comparing differentially expressed transcripts in apical buds of spurs sampled on adult ‘Gala’ apple trees, between ‘ON’ (fruited) and ‘OFF’ (deflowered) trees. In apical buds of ‘ON’ trees, more likely to be in vegetative state, transcripts highlighted responses to stresses such as starvation for sucrose and phosphates, stress hormonal signaling and down-regulated cell detoxification processes, whereas transcripts homologous to genes known as repressor of flowering induction (such as *TEMPRANILLO 1*) and involved in SAM maintenance in the vegetative state (such as *KNAT* and *NAM*) were up-regulated. In apical buds of ‘OFF’ trees, more likely to be induced to flower, transcripts showed active cell biogenesis and detoxification, with several promoters of floral induction (such as *FTM1* and *SPL* transcripts) up-regulated. Timing and transitions stages from vegetative state (Insert **a**) to domed meristem (stage 2, from day 203, Insert **b**) and floral meristem (State 4, Insert **c**), as defined by Foster et al. [29] are exemplified by photos of histological longitudinal sections in apical buds of ‘Gala’ apple trees

In this experiment, apical buds of ‘ON’ trees exhibited differential expression in transcripts involved in metabolic processes, which suggest that they could be starved and under oxidative stress (Fig. 7). Depletion in carbohydrate supply in apical buds, which is one of the earliest observed events, is likely to result from high crop load. Even though the presence of fruit has been shown to enhance photosynthesis in adjacent leaves [114], the competition for carbohydrates between vegetative and reproductive growth is a well-known and documented phenomenon [69, 90]. The necessary balance between vegetative and reproductive growth, as well as the requirement for a minimum number of leaves for fruit set and development to take place, was suggested by Huet [39] and Nielsen and Dennis [72], who demonstrated that the FI rate increases with the number of leaves associated with the bourse in apple and pear. However, if the autonomous pathway is described as the number of leaves necessary to induce flowering [77], it will be almost impossible to distinguish this effect from that of carbohydrate availability due to the corresponding photosynthetic activity of leaves. The source/sink relationships and genetic control of precise cell territories will need further investigation to explain the diversity of bud fates and topology that are observed at a more integrated plant scale [20].

In addition, the influence of carbohydrate allocation to the development of specific tissues or organs could also preside over “go/no go” decisions such as organ drop, SAM death or FI triggering. Indeed, carbohydrates are considered not only as nutrients but also as signaling molecules [32], and the signaling role of starch and sucrose metabolism in FI has been proposed for a long time [7, 18]. In our study, there was no evidence of a differential expression of transcripts involved in primary carbohydrate metabolism between apical buds of ‘ON’ and ‘OFF’ trees; however, large differences in expression were identified in genes encoding enzymes that control secondary metabolism, including phosphatase-reductases, glycosyl-transferases, cellulose synthases, which were all down-regulated in apical buds of ‘ON’ trees. This suggests that, even though active photosynthesis can be assumed in ‘ON’ trees bearing heavy crop, carbon may not be enough available to apical tissues, and could lead to local carbon depletion and reduced cellular activity. Indeed, the maintenance of cell division and differentiation has been shown to generate a strong carbohydrate requirement in SAM [27]. The up-regulation in ‘ON’ trees of two transcription factors involved in SAM maintenance (*KNAT1* and *NAM*) is consistent with this assumption. *KNAT1*, a member of the KNOX family [85] and *NAM* [92], which is involved in the SAM constitution during embryogenesis, are both involved in SAM patterning and prevent meristematic cell differentiation. Therefore their up-regulation in ‘ON’ trees is consistent with a reduction in carbon demand, FI inhibition and maintenance of vegetative fate in SAM. Moreover, two genes known to be involved in the control of axillary bud, *MAX1* and *BRC1*, were also up-regulated in ‘ON’ trees. The up-regulation of *MAX1*, which is an inhibitor of AM development and branching [9] is consistent with antagonism between vegetative growth and fruit load. *BRC1*, a homologue of the maize *TEOSINTE BRANCHED1*, is also known to be involved in control of axillary branching [11]. Interestingly, *BRC1* has been also shown to interact with *FT* and *TFL1* in Arabidopsis during the floral transition of axillary meristems [73].

The link between nutritional and redox status is known to be mediated by the circadian clock that enables daily regulation of cell metabolism, energy balance and redox status [37]. In our study, several genes involved in the control of oxidative stress tolerance, cellular ion homeostasis, cellular redox status and senescence were up-regulated in apical buds of ‘ON’ trees. This suggests that ROS could accumulate in these buds. In Arabidopsis, the transition from vegetative to reproductive phase has been associated with enhanced accumulation of oxidized proteins [46] and a mutant lacking thylakoid ascorbate peroxidase and cytosolic ascorbate peroxidase1 exhibited an early bolting phenotype [66]. It has thus been suggested that plants subjected to stress flower earlier and that there might be a commensurate response to stress intensity, stepping up from a strategy based on tolerating the effects of stress, for instance through OXS expression, to stress escape via reproduction [8]. However, depending on the species both delayed and early flowering have been observed under stress conditions and the decision on FI triggering may differ between annual and perennial plants. Further characterization of the putative link between cell redox status in SAM and flowering could contribute to deciphering the physiological mechanisms involved in FI in adult apple trees.

### The hormones could contribute to the decision to flower

The hormonal responses differentially expressed between ‘ON’ and ‘OFF’ trees appeared consistent with the nutritional and oxidative stress perceived by apical buds in ‘ON’ trees compared with that in ‘OFF’ trees. In particular, the redox hub activity is linked to hormonal signaling and results in plant resistance to biotic and abiotic stresses. For instance, in addition to its antioxidant function, ascorbic acid is required for the biosynthesis of the plant hormones abscisic acid, GA, and ethylene [3, 4]. Moreover, low amounts of ascorbic acid promote the accumulation of the phytoalexin camalexin [17] as well as salicylic acid [6] and this has been demonstrated to influence flowering time [63]. As genes and transcription factors induced by brassinosteroid and auxin were also differentially expressed in our study, most hormonal functions were differentially oriented between ‘ON’ and ‘OFF’ trees.

Among them, GA biosynthesis represents the strongest candidate function for its direct involvement in the regulation of flowering control in apple, even though the roles of the different forms of GA have not so far been elucidated. Application of GA to apple trees has showed that GA_7_ has the most inhibitory effect on FI [102], and horticultural practices commonly involve the application of GA during ‘OFF’ years to prevent an excessive FI and thus attenuate the biennial bearing cycle [84]. Hence, bioactive GAs appear to have an inhibitory effect on key flowering genes/steps in apple, and are considered to have an opposite effect on FI in perennial plants, compared with their role in annual plants [42]. It is noteworthy that in our study several differentially expressed transcripts involved in the GA biosynthesis pathway were identified in QTL cluster intervals that control tree production and alternation: *MdGA20ox1a* and *MdGA3ox-like-b* on LG1 and *MdGA2ox8a* on LG10 [33]. In addition, two copies of *GA2ox* and one copy of *GA20ox*, which contribute to catabolism and metabolism of active GA forms, respectively, were up-regulated in apical buds of ‘ON’ trees. These results reinforce the assumption that, in apple, bioactive GAs may play a role in the control of key flowering genes/steps.

### Putative flowering genes and floral repressors involved in FI in adult apple trees

Transcripts with sequence homology to major genes known as key actors in control of FI were differentially expressed in apical buds of adult apple trees: two were floral repressors (*TEM1 and AGL31*/*FLC-Like*) that were up-regulated in ‘ON’ trees, whereas genes enhancing flowering such as *SPL* genes were up-regulated in ‘OFF’ trees.

In our experiment, *TEM1* was up-regulated in ‘ON’ trees at both day 131 and 151. This gene belongs to the complex *RAV* family of transcription factors [14, 65, 74], in which there may be functional redundancy, since both *TEM1* and *TEM2* act as direct repressors of the *FT* gene. *TEM1* transcription is induced when leaf senescence is accelerated by phytohormones such as ethylene or methyl jasmonate [1, 40] and *TEM1* has been described as an integrator of different flowering pathways (age dependent, GA, light quality and intensity, ambient temperature and brassinosteroids) [65]. *TEM1* has been shown to be expressed in vascular and mesophyll tissues in leaves although the levels and spatial distribution change through development [14]. In the present study, we found that *TEM1* is expressed in apical buds at early stages after full bloom (day 131 and 151) and with differential expression levels consistent with an inhibitory role on FI in ‘ON’ trees. As *TEM1* and *TEM2* exhibit a circadian-clock dependent expression profile with a maximum at midafternoon [14], further investigations on the diurnal pattern of *TEM1* expression in apple tree apical buds should be performed before drawing any conclusions.

Up-regulation in ‘ON’ trees of the transcription factor *MAF2* (*MADS AFFECTING FLOWERING 2*), which belongs to a large *MADS-box* family of flowering repressors was found, but at day 222 only, i.e. after the normal FI period. In Arabidopsis, *MAF2* is known to be homologous to *FLOWERING LOCUS C* and *M* (*FLC* and *FLM*) and to be involved in the vernalization pathway and in the adaptation of flowering time of *Arabidopsis thaliana* to cold temperatures [97]. Since *FLC* and *FLC-like* transcription factors have been reported to be up-regulated by oxidized glutathione [49], the late expression of *MAF2* could result from the redox status in apical buds of ‘ON’ trees. Similarly, the differential expression of the *PSEUDO RESPONSE REGULATOR* (*PRR*) protein *PRR5*, which was observed in this experiment and which is known to encode components of the circadian clock in *Arabidopsis thaliana* [81] might be linked to the daily cellular detoxification and regulation of redox status. However, these suggestions will need further investigations.

Other putative actors in our system were *SQUAMOSA PROMOTER BINDING-LIKE* (*SPL*) transcription factors. In Arabidopsis, expression of both *SPL5* and *SPL9* is regulated by the microRNA *miR156*. However, in our experiment *miR156* did not show differential expression between ‘ON’ and ‘OFF’ trees, consistently with the up-regulation of *SPL-like* genes found in citrus without differential expression of *miR156* [88]. However, the role of *miR156* in floral induction will need further investigations since *miR156* abundance is reduced with plant age and is associated with vegetative phase transition in *Arabidopsis thaliana* [113]*.* In our data, *SPL5* and *SPL9* were up-regulated in ‘OFF’ trees at day 222 only and had similar expression patterns to those of *MdAP1a* and *MdAP1b*. This could be in line with *SPL9* role in promoting the expression of *AP1* in Arabidopsis [109], but as a late event in the FI process. Interestingly, recent results highlighted interactions between GA and *SPL* transcription factors in SAM of *Arabidopsis thaliana* under long days [2, 75]. These authors showed that GA is required in vascular tissues to increase the levels of *FT* and *TWIN SISTER OF FT* (*TSF*) mRNAs which encode a systemic signal transported from the leaves to SAM during FI. They suggest that GA might have spatially separated functions between leaves and SAM since GA could facilitate the activation of *FT* in leaves and *SPL* genes in the meristem. In addition, Torti et al*.* [101] described a *FT*-independent pathway, in which *FTM1* was expressed in SAM under long days. Our results could be consistent with this scheme, since *FTM1* transcript was differentially expressed at the time of floral bud initiation (day 151) and afterwards, until flower meristem differentiation (day 222). Even though it is impossible to speculate on *FT* involvement in FI triggering since leaves were not considered in this experiment and despite *SHORT VEGETATIVE PHASE* (*SVP*), which could be suspected to be present, was not differentially expressed in the microarray, several major genes were present and differentially expressed in apical buds between ‘ON’ and ‘OFF’ apple trees: *GA2ox*, *FTM1*, *SPL5* and *SPL9*.

## Conclusion

The transcripts differentially expressed between inductive (‘OFF’) and non-inductive (‘ON’) conditions in adult apple trees suggests that different flowering pathways could be involved in the control of FI in adult apple trees. Changes in the nutritional and redox status could be part of the autonomous pathway, whereas GA (through *GA20ox* and *GA2ox*) and circadian clock (through *PRR5*) and ambient temperature (through *FLC-Like*) pathways could be involved as well. The main hypothesis emerging from our results is that unfavorable redox and nutritional status of buds could induce hormonal response that may in turn activate key regulators of meristem fate. However, at that stage causes and effects cannot be inferred from the data. In addition, we found that key regulators of stress signaling (LRR-receptor-like and protein kinase) and of meristem transition in *Arabidopsis thaliana* (*NO APICAL MERISTEM* and *SPL9*) were present in QTL intervals for traits linked to biennial bearing in apple. Studying allelic diversity of the apple tree genes and comparing them to the phenotypic behavior can yield powerful tools for use in breeding regular varieties using molecular markers.

## Availability of data and materials

Microarray samples were deposited in NCBI’s Gene Expression Omnibus [5] and are accessible through GEO Series accession number GSE64646 (http://www.ncbi.nlm.nih.gov/geo/query/acc.cgi?token=onsdgqwotbuhbox&acc=GSE64646). Normalized data along with the R script used for analyzing the data were deposited on GitHub (https://github.com/Baptiste-Guitton/Microarray_biennial_bearing_apple_Gala) that enable the reproducibility of the analyses.

## Additional files

Additional file 1: Figure S1. Apple bud sampling strategy. (PDF 130 kb) Additional file 2: Table S1. List of genes and primers used for real-time quantitative PCR analyses. **Table S2.** Genomic position of the genetic markers of the ‘Starkrimson’ x ‘Granny Smith’ apple genetic map [35]. **Table S3.** Genomic position of the QTL related to biennial bearing using the ‘Starkrimson’ x ‘Granny Smith’ apple genetic map [25, 35]. **Table S4.** Significance of the effect estimated by ANOVA for each apple gene studied by qRT‐PCR. **Table S5.** Significance of the difference of relative expression between apple trees inhibiting flowering (‘ON’) and trees initiating flowering (‘OFF’) for each studied gene and at each date. **Table S6.** Number of genes, mean and standard error per group of genes with a differential expression at least one time of the three sampled dates. **Table S7.** Gene ontology (GO) categorization of genes differentially expressed in apical buds of apple trees inhibiting flowering (‘ON’) or initiating flowering (‘OFF’) at day 131 (A), 151 (B) and 222 (C). **Table S8.** List of apple genes showing significant differential expression at day 131 (A), 151 (B) and 222 (C). **Table S9.** Genomic position of the genes expressed differentially in apple trees inhibiting flowering (‘ON’) and trees initiating flowering (‘OFF’) on the ‘Golden Delicious’ genome. **Table S10.** List of genes showing significant differential expression between apple trees inhibiting flowering (‘ON’) and trees initiating flowering (‘OFF’) at least one of the three dates of the experiment and located within QTL intervals for traits related to biennial bearing using the ‘Starkrimson’ x ‘Granny Smith’ genetic map [25, 35]. (XLS 1000 kb) Additional file 3: Figure S2. Expression pattern of *MdFT1*, *MdFT2*, *AFL1*, *AFL2*, *MdAP1a*, *MdAP1b*, *MdSOC1-like* and *MdTFL1* during the growing season in ‘Gala’ apple trees measured by quantitative real-time PCR using spur apical buds harvested at day 118, 131, 151, 180 and 222. (PDF 61 kb) Additional file 4: Figure S3. Validation of microarray relative expression (log2 ratio) by expression of selected candidate genes by qRT-PCR. (PDF 63 kb) Additional file 5: Figure S4. Hierarchical tree graph of over-represented Gene Ontology (GO) terms in biological process category generated by Single Enrichment Analysis (SEA) for differentially expressed apple genes at day 131 (A), 151 (B) and 222 (C). (PDF 533 kb) Additional file 6: Figure S5. Physical position on the apple reference genome of QTL related to biennial bearing and genes significantly differentially expressed. (PNG 1549 kb)
