# Supplementary material for: Analysis of transcripts differentially expressed between fruited and deflowered ‘Gala’ adult trees: a contribution to biennial bearing understanding in apple
Source: BMC Plant Biol. 2016 Feb 29;16:55. doi: 10.1186/s12870-016-0739-y (PMC4770685; doi:10.1186/s12870-016-0739-y)
Supplement: Additional file 1: Figure S1. — Apple bud sampling strategy. (PDF 130 kb) [file 12870_2016_739_MOESM1_ESM.pdf]

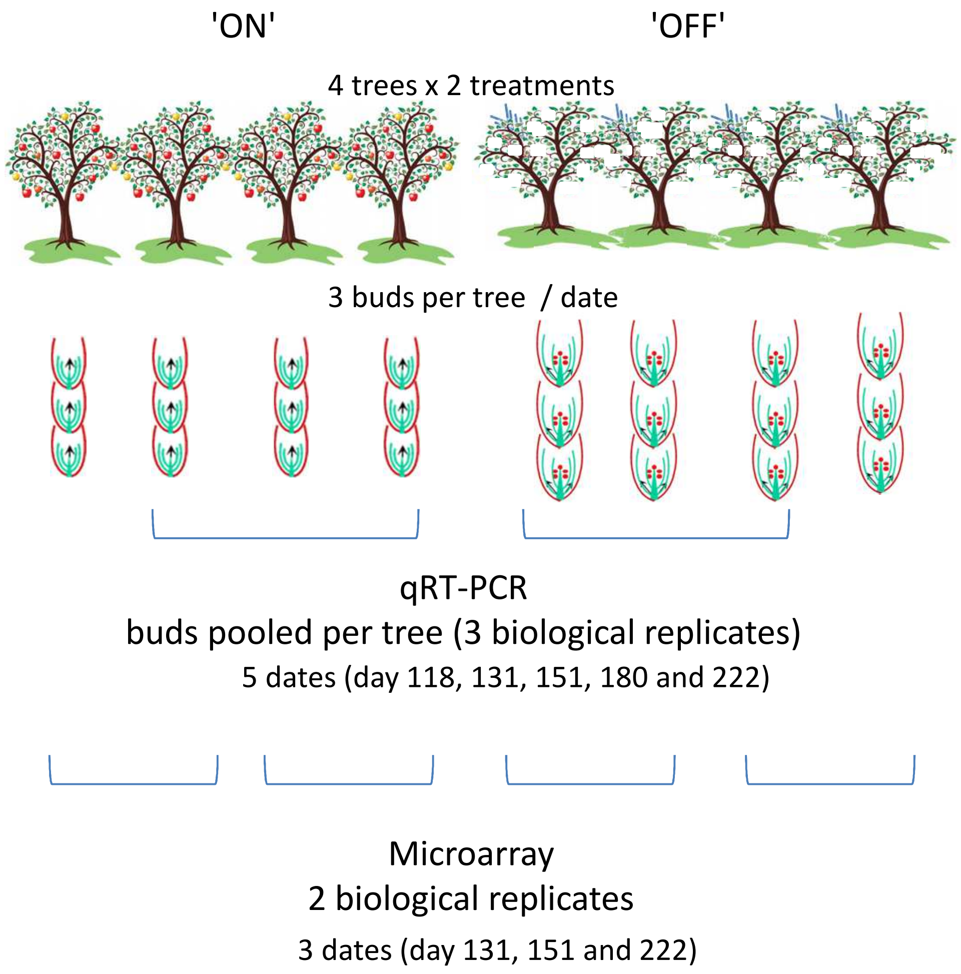

Additional file 1: Figure S1. Apple bud sampling strategy.

Terminal buds of spurs were harvested between 1000 h and 1200 h from April to August 2010, at day 118, 131, 151, 180 and 222. Four ‘Gala’ trees per treatment ('ON' - fruited and 'OFF' - deflowered trees) and three buds per tree were sampled at each harvest date. Three trees and three spur apical buds per tree over the five time points were used for mRNA profiling by qRT-PCR. Then, three dates among the five were chosen for microarray analyses, and on each of these dates, three buds from the four sampled trees were used.
