# Supplementary material for: Analysis of transcripts differentially expressed between fruited and deflowered ‘Gala’ adult trees: a contribution to biennial bearing understanding in apple
Source: BMC Plant Biol. 2016 Feb 29;16:55. doi: 10.1186/s12870-016-0739-y (PMC4770685; doi:10.1186/s12870-016-0739-y)
Supplement: Additional file 3: Figure S2. — Expression pattern of MdFT1, MdFT2, AFL1, AFL2, MdAP1a, MdAP1b, MdSOC1-like and MdTFL1 during the growing season in ‘Gala’ apple trees measured by quantitative real-time PCR using spur apical buds harvested at day 118, 131, 151, 180 and 222. (PDF 61 kb) [file 12870_2016_739_MOESM3_ESM.pdf]

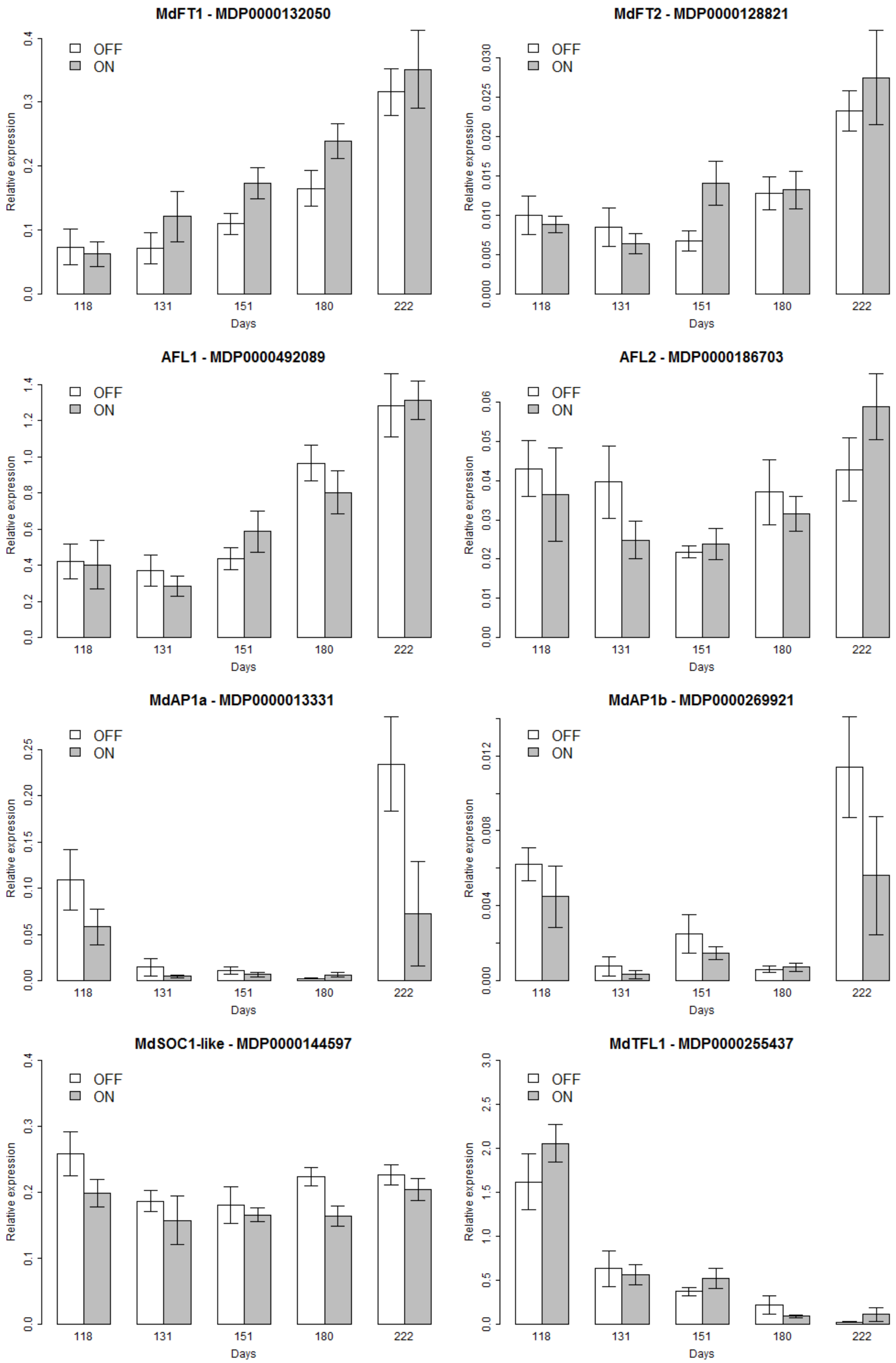
MdFT1 - MDP0000132050
OFF
ON
Relative expression
0.0
0.1
0.2
0.3
0.4
118
131
151
180
222
Days
MdFT2 - MDP0000128821
OFF
ON
Relative expression
0.000
0.005
0.010
0.015
0.020
0.025
0.030
118
131
151
180
222
Days
AFL1 - MDP0000492089
OFF
ON
Relative expression
0.0
0.2
0.4
0.6
0.8
1.0
1.2
1.4
118
131
151
180
222
Days
AFL2 - MDP0000186703
OFF
ON
Relative expression
0.00
0.01
0.02
0.03
0.04
0.05
0.06
118
131
151
180
222
Days
MdAP1a - MDP0000013331
OFF
ON
Relative expression
0.00
0.05
0.10
0.15
0.20
0.25
118
131
151
180
222
Days
MdAP1b - MDP0000269921
OFF
ON
Relative expression
0.000
0.004
0.008
0.012
118
131
151
180
222
Days
MdSOC1-like - MDP0000144597
OFF
ON
Relative expression
0.0
0.1
0.2
0.3
0.4
118
131
151
180
222
Days
MdTFL1 - MDP0000255437
OFF
ON
Relative expression
0.0
0.5
1.0
1.5
2.0
2.5
3.0
118
131
151
180
222
Days

Figure S2. Expression pattern of *MdFT1*, *MdFT2*, *AFL1*, *AFL2*, *MdAP1a*, *MdAP1b*, *MdSOC1-like* and *MdTFL1* during the growing season in 'Gala' apple trees measured by quantitative real-time PCR using spur apical buds harvested at day 118, 131, 151, 180 and 222.

Comparison was made between expression levels of spur apical buds of apple trees bearing no crop ('OFF'), heavy crop ('ON') in 2010. Graphics represent the average of the nine data points per date per treatment with associated standard deviation. Relative expression was calculated using the *α-ACTIN* housekeeping gene.
