# Supplementary material for: Analysis of transcripts differentially expressed between fruited and deflowered ‘Gala’ adult trees: a contribution to biennial bearing understanding in apple
Source: BMC Plant Biol. 2016 Feb 29;16:55. doi: 10.1186/s12870-016-0739-y (PMC4770685; doi:10.1186/s12870-016-0739-y)
Supplement: Additional file 4: Figure S3. — Validation of microarray relative expression (log2 ratio) by expression of selected candidate genes by qRT-PCR. (PDF 63 kb) [file 12870_2016_739_MOESM4_ESM.pdf]

Figure S3 : Validation of microarray relative expression (log2 ratio) by expression of selected candidate genes by RT-qPCR.

MDP number	Gene annotation	Day	Treatment	qPCR relative expression to *HistoneH3*	Microarray relative expression (log2 ratio)
MDP0000492089	*AFL1*	131	OFF	0.311983	3.7
MDP0000492089	*AFL1*	151	OFF	0.302650	3.95
MDP0000492089	*AFL1*	222	OFF	1.011256	4.57
MDP0000492089	*AFL1*	131	ON	0.258740	3.38
MDP0000492089	*AFL1*	151	ON	0.376367	3.68
MDP0000492089	*AFL1*	222	ON	0.946711	4.48
MDP0000186703	*AFL2*	131	OFF	0.026533	3.25
MDP0000186703	*AFL2*	151	OFF	0.015800	3.08
MDP0000186703	*AFL2*	222	OFF	0.032500	3.75
MDP0000186703	*AFL2*	131	ON	0.020960	3.09
MDP0000186703	*AFL2*	151	ON	0.016925	2.83
MDP0000186703	*AFL2*	222	ON	0.034538	3.91
MDP0000013331	*MdAP1a*	131	OFF	0.007789	-0.09
MDP0000013331	*MdAP1a*	151	OFF	0.006417	0.61
MDP0000013331	*MdAP1a*	222	OFF	0.189250	3.55
MDP0000013331	*MdAP1a*	131	ON	0.006136	-0.17
MDP0000013331	*MdAP1a*	151	ON	0.003916	-0.11
MDP0000013331	*MdAP1a*	222	ON	0.088950	2.37
MDP0000269921	*MdAP1b*	131	OFF	0.000505	0.44
MDP0000269921	*MdAP1b*	151	OFF	0.001337	1.32
MDP0000269921	*MdAP1b*	222	OFF	0.008507	3.5
MDP0000269921	*MdAP1b*	131	ON	0.000094	0.46
MDP0000269921	*MdAP1b*	151	ON	0.001116	0.85
MDP0000269921	*MdAP1b*	222	ON	0.006606	2.58
MDP0000132050	*MdFT1*	131	OFF	0.003238	3.44
MDP0000132050	*MdFT1*	151	OFF	0.004791	3.73
MDP0000132050	*MdFT1*	222	OFF	0.011980	4.52
MDP0000132050	*MdFT1*	131	ON	0.003745	3.45
MDP0000132050	*MdFT1*	151	ON	0.005926	4.35
MDP0000132050	*MdFT1*	222	ON	0.009844	4.39
MDP0000128821	*MdFT2*	131	OFF	0.000283	2.52
MDP0000128821	*MdFT2*	151	OFF	0.000304	2.79
MDP0000128821	*MdFT2*	222	OFF	0.000922	3.7
MDP0000128821	*MdFT2*	131	ON	0.000300	2.73
MDP0000128821	*MdFT2*	151	ON	0.000548	3.19
MDP0000128821	*MdFT2*	222	ON	0.001277	3.67
MDP0000255437	*MdTFL1*	131	OFF	0.023282	2.95
MDP0000255437	*MdTFL1*	151	OFF	0.014114	2.55
MDP0000255437	*MdTFL1*	222	OFF	0.000910	0.39
MDP0000255437	*MdTFL1*	131	ON	0.025090	2.97
MDP0000255437	*MdTFL1*	151	ON	0.016196	2.78
MDP0000255437	*MdTFL1*	222	ON	0.005751	0.96

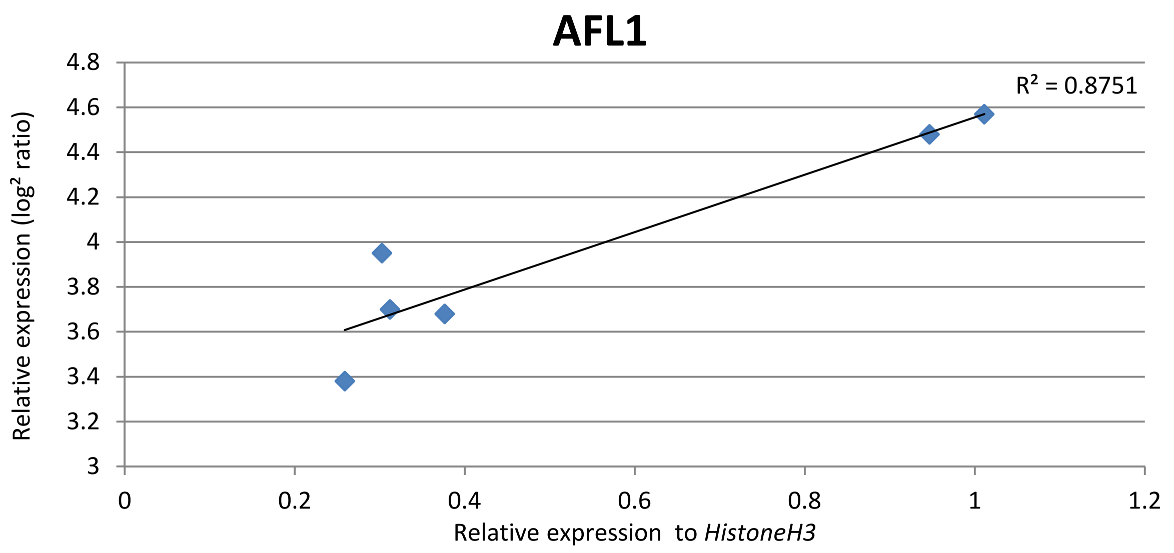
AFL1
R² = 0.8751
4.8
4.6
4.4
4.2
4
3.8
3.6
3.4
3.2
3
Relative expression (log² ratio)
0
0.2
0.4
0.6
0.8
1
1.2
Relative expression to HistoneH3

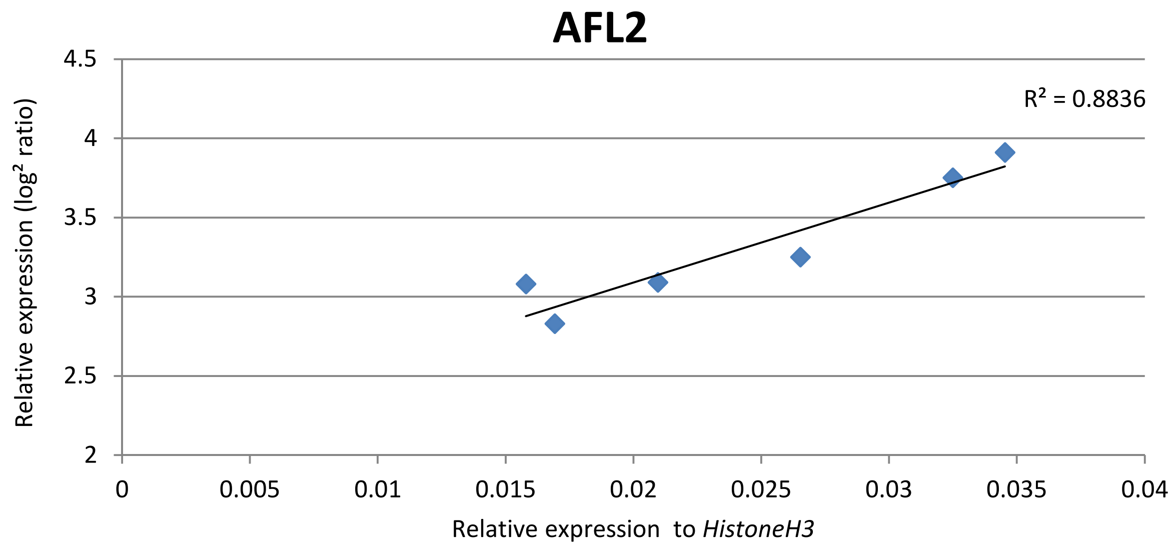
AFL2
R² = 0.8836
4.5
4
3.5
3
2.5
2
Relative expression (log² ratio)
0
0.005
0.01
0.015
0.02
0.025
0.03
0.035
0.04
Relative expression to HistoneH3

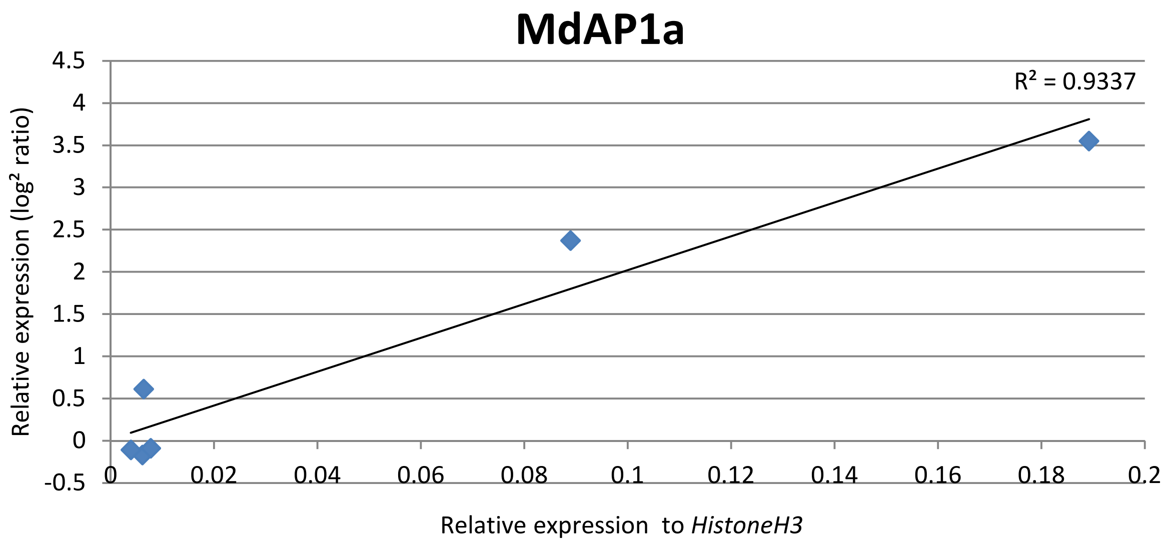
MdAP1a
R² = 0.9337
4.5
4
3.5
3
2.5
2
1.5
1
0.5
0
-0.5
Relative expression (log² ratio)
0
0.02
0.04
0.06
0.08
0.1
0.12
0.14
0.16
0.18
0.2
Relative expression to HistoneH3

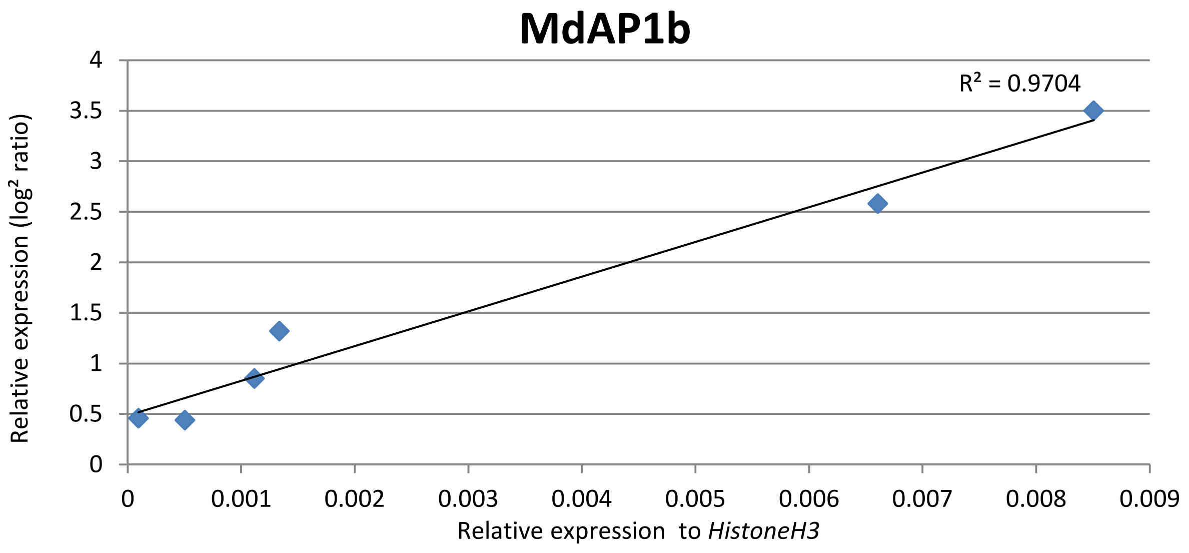

MdAP1b
R² = 0.9704
Relative expression (log² ratio)
0
0.5
1
1.5
2
2.5
3
3.5
4
0
0.001
0.002
0.003
0.004
0.005
0.006
0.007
0.008
0.009
Relative expression to HistoneH3

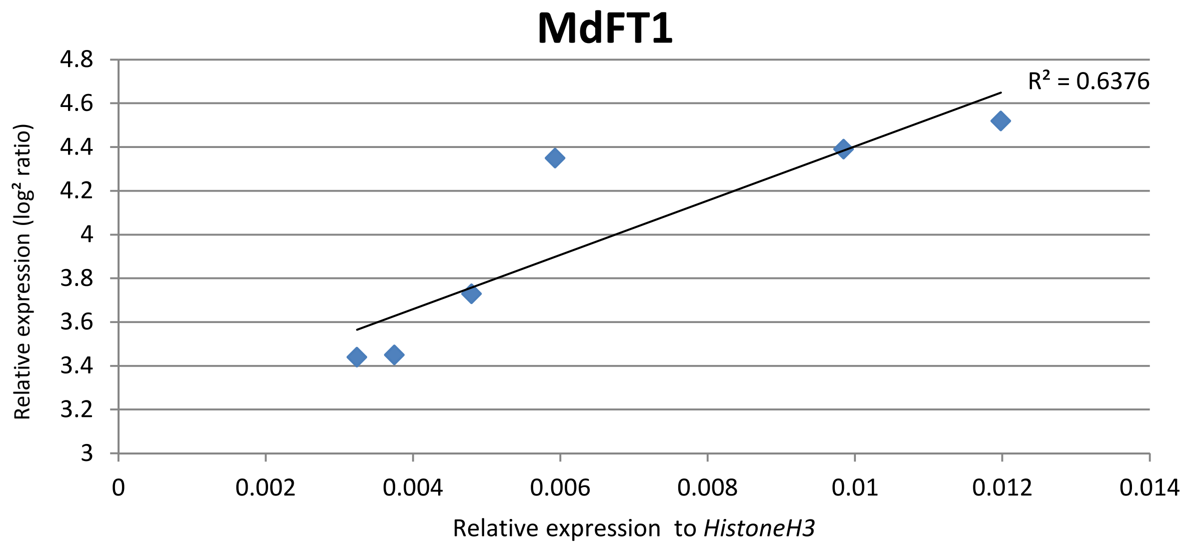

MdFT1
R² = 0.6376
Relative expression (log² ratio)
3
3.2
3.4
3.6
3.8
4
4.2
4.4
4.6
4.8
0
0.002
0.004
0.006
0.008
0.01
0.012
0.014
Relative expression to HistoneH3

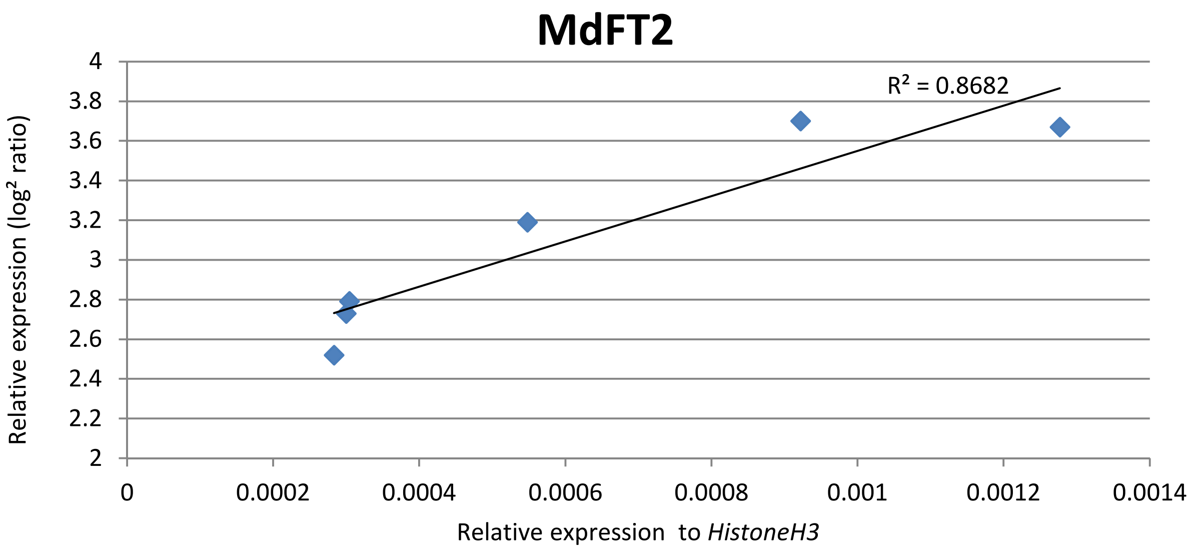

MdFT2
R² = 0.8682
Relative expression (log² ratio)
2
2.2
2.4
2.6
2.8
3
3.2
3.4
3.6
3.8
4
0
0.0002
0.0004
0.0006
0.0008
0.001
0.0012
0.0014
Relative expression to HistoneH3

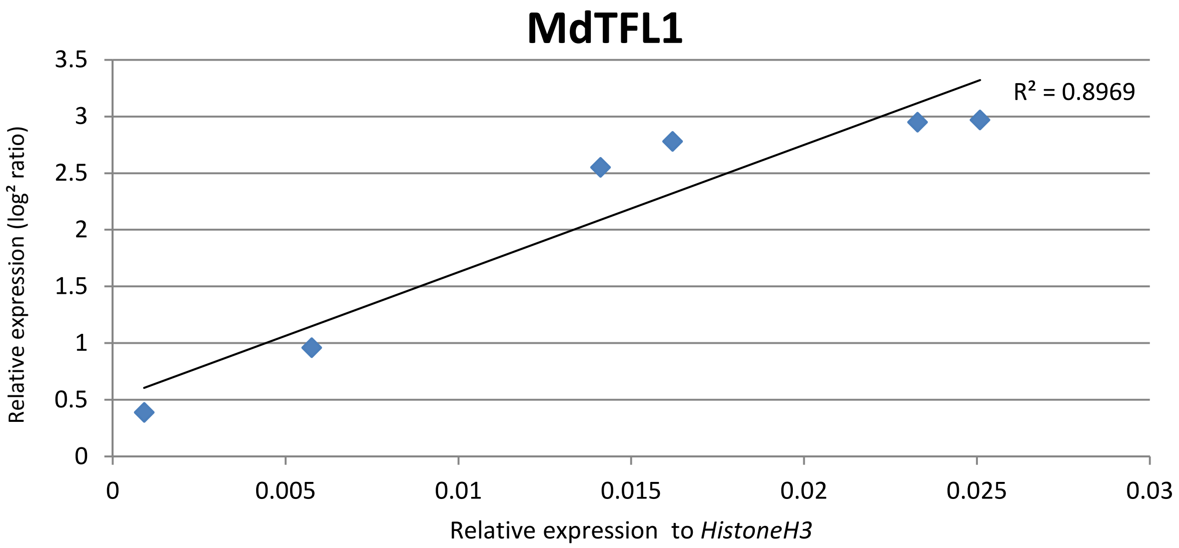

MdTFL1
3.5
3
2.5
2
1.5
1
0.5
0
Relative expression (log² ratio)
R² = 0.8969
0
0.005
0.01
0.015
0.02
0.025
0.03
Relative expression to HistoneH3
