# Supplementary material for: Analysis of transcripts differentially expressed between fruited and deflowered ‘Gala’ adult trees: a contribution to biennial bearing understanding in apple
Source: BMC Plant Biol. 2016 Feb 29;16:55. doi: 10.1186/s12870-016-0739-y (PMC4770685; doi:10.1186/s12870-016-0739-y)
Supplement: Additional file 5: Figure S4. — Hierarchical tree graph of over-represented Gene Ontology (GO) terms in biological process category generated by Single Enrichment Analysis (SEA) for differentially expressed apple genes at day 131 (A), 151 (B) and 222 (C). (PDF 533 kb) [file 12870_2016_739_MOESM5_ESM.pdf]

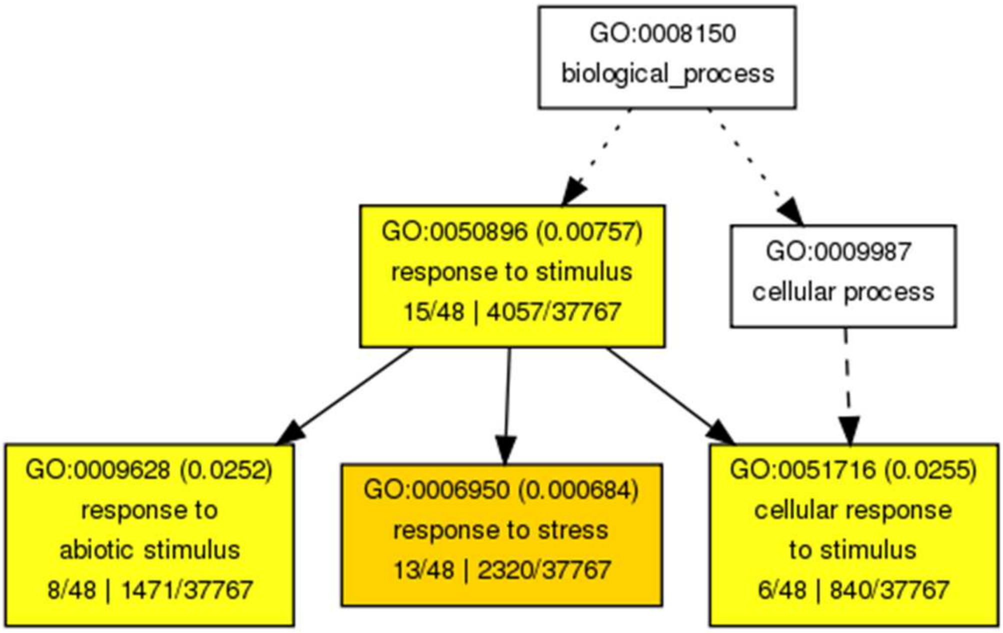

Additional file 5: Figure S4A. Hierarchical tree graph of over-represented Gene Ontology (GO) terms in biological process category generated by Single Enrichment Analysis (SEA) for differentially expressed apple genes at day 131.

Boxes in the graph contain GO identification, GO term label and for significant terms statistical information is displayed (p-value, number of gene in the query list of the present GO/total number of gene in the query list and number of genes in the present GO in the reference genome/number of annotated genes in the reference genome). The significant GO terms (adjusted $P \leq 0.05$) are colored, while non-significant terms are not.

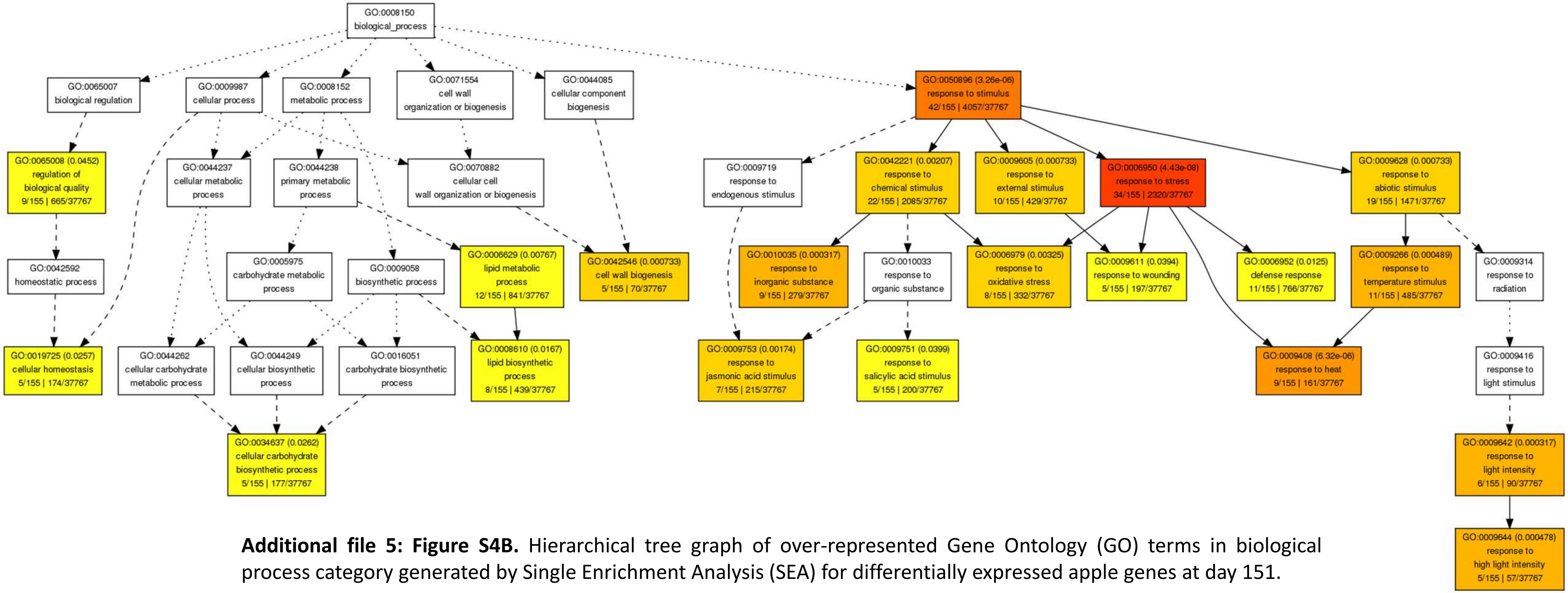

Additional file 5: Figure S4B. Hierarchical tree graph of over-represented Gene Ontology (GO) terms in biological process category generated by Single Enrichment Analysis (SEA) for differentially expressed apple genes at day 151.

Boxes in the graph contain GO identification, GO term label and for significant terms statistical information is displayed (p-value, number of gene in the query list of the present GO/total number of gene in the query list and number of genes in the present GO in the reference genome/number of annotated genes in the reference genome). The significant GO terms (adjusted $P \leq 0.05$) are colored, while non-significant terms are not.

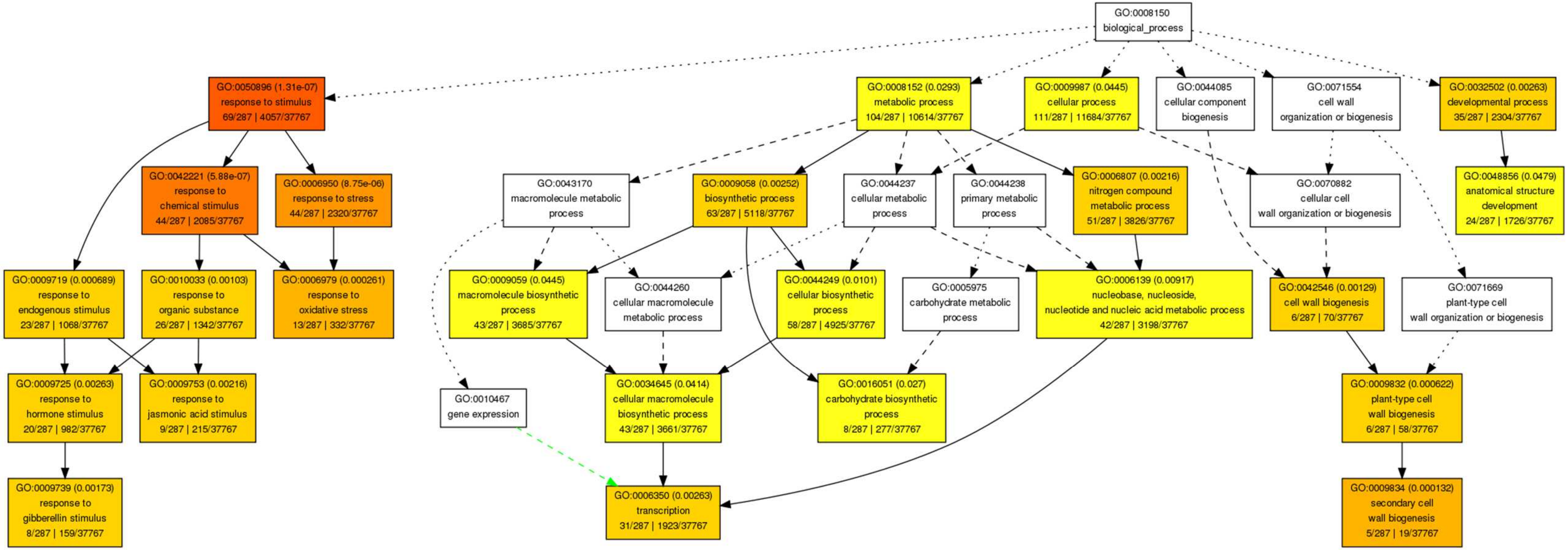

Additional file 5: Figure S4C. Hierarchical tree graph of over-represented Gene Ontology (GO) terms in biological process category generated by Single Enrichment Analysis (SEA) for differentially expressed apple genes at day 222.

Boxes in the graph contain GO identification, GO term label and for significant terms statistical information is displayed (p-value, number of gene in the query list of the present GO/total number of gene in the query list and number of genes in the present GO in the reference genome/number of annotated genes in the reference genome). The significant GO terms (adjusted $P \leq 0.05$) are colored, while non-significant terms are not.
