# Supplementary figures and images for: Analysis of transcripts differentially expressed between fruited and deflowered ‘Gala’ adult trees: a contribution to biennial bearing understanding in apple
Source: BMC Plant Biol. 2016 Feb 29;16:55. doi: 10.1186/s12870-016-0739-y (PMC4770685; doi:10.1186/s12870-016-0739-y)

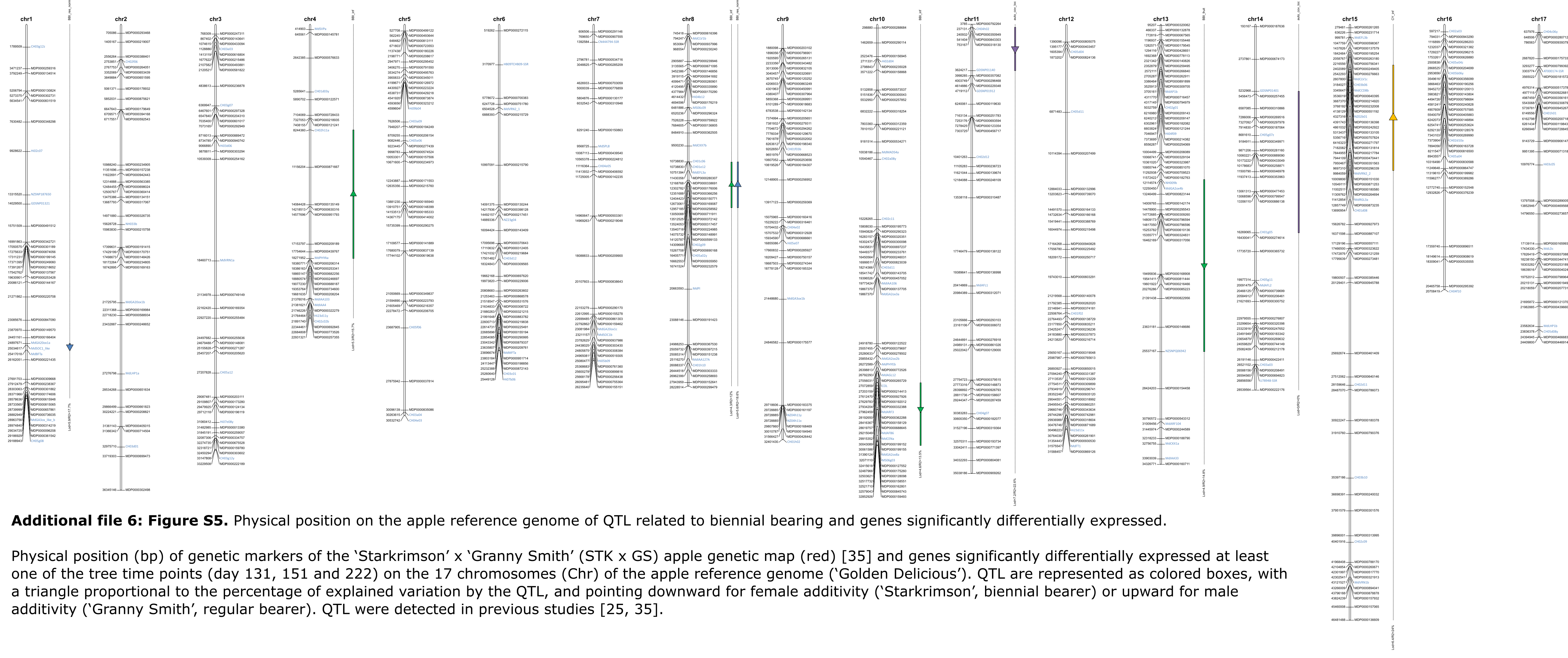

Supplement: Additional file 6: Figure S5. — Physical position on the apple reference genome of QTL related to biennial bearing and genes significantly differentially expressed. (PNG 1549 kb) [file 12870_2016_739_MOESM6_ESM.png]
